# Offline User Authentication Ensuring Non-Repudiation and Anonymity

**DOI:** 10.3390/s22249673

**Published:** 2022-12-10

**Authors:** Ya-Fen Chang, Wei-Liang Tai, Ka-Ho Fung

**Affiliations:** 1 Department of Computer Science and Information Engineering, National Taichung University of Science and Technology, Taichung 404, Taiwan; 2 Bachelor Degree Program of Artificial Intelligence, National Taichung University of Science and Technology, Taichung 404, Taiwan

**Keywords:** offline, non-repudiation, anonymity, authentication, security

## Abstract

User authentication is the key to ensuring that only authorized users can deal with specific affairs and access services. Applications or systems possessing different properties or requirements need different authentication schemes. For example, some institutions or companies need executives to manage or inspect their corresponding departments while the inspected department should not know who the executives are but only can verify their legitimacy. This paper designs a non-repudiation and anonymity-ensured user authentication system to meet the mentioned special requirements. We also propose a user authentication scheme to ensure that the designed system can work as claimed. In the system, a department is equipped with an authentication device, namely the department authentication device, to authenticate an executive while the executive’s identity is not revealed to the department and only the department’s authentication device can identify the executive for non-repudiation. An executive is equipped with an authentication device to have himself/herself authenticated by the department’s authentication device. Moreover, authentication data stored in an executive’s authentication device does not need to be updated even when management personnel changes are made.

## 1. Introduction

The purpose of user authentication is to verify that a user is indeed the claimed one. That is, it must be able to identify a user uniquely, and there must exist a way to unambiguously verify the legitimacy of the user. In our daily life, user authentication is common. For example, when a user wants to open a savings account, he/she needs to provide his/her identity card, health insurance card, or driving license to prove who he/she is. Additionally, a user authentication system ensures that only authorized users can deal with specific affairs and access services. This property makes user authentication essential because institutions, companies, or organizations need to protect their resources and ensure that only legal staff or members can deal with specific affairs or access required services.

Factors for user authentication can be classified into three types: something held, something embodied, and something known. Something held can be a barcode, a QR code, a magnetic card, an IC card, a smart card, or any physical object possessed by a user. Something embodied can be divided into three categories. First, it can be any biological feature, such as a user’s DNA or blood. Second, it can be a user’s morphological features, such as fingerprints, hand geometry, or iris patterns. Third, it can be behavioral characteristics of a user such as how the user speaks, walks, or types on a keyboard. Something known means the specific knowledge only known by the user, such as a password or PIN. In some applications, the system issues a user a mobile device or smart card that can store parameters and compute required parameters. When the user wants to access the system, he/she needs to use the issued mobile device or smart card to prove his/her legality to the system. Some authentication schemes adopting mobile devices or smart cards to enhance security have been proposed [1,2,3,4,5,6,7,8,9,10,11,12,13,14,15,16,17,18,19].

Applications or systems possessing different properties or requirements need different authentication schemes. For example, some institutions or companies need executives to manage or inspect their corresponding departments while the inspected department should not know who the executives are but only can verify their legitimacy. On the other hand, some users care about personal privacy such that they do not want to be traced. In such cases, anonymity needs to be ensured. In conventional authentication schemes, a user’s identity will be adopted to identify the user. However, this approach means these authentication schemes lack anonymity. Different from conventional authentication schemes, anonymity-ensured authentication schemes allow a user to be authenticated without revealing his/her real identity. The most significant advantage of these anonymous authentication schemes is to protect the user’s identity and prevent other users from tracking and identifying a specific user. To comply with this special requirement, several schemes ensuring anonymity have been proposed [5,6,7,8,9,10,11,12,13,14,15,16,17,18,19].

On the other hand, non-repudiation is another important security requirement in many applications. It denotes that a user cannot deny what he/she has done. To ensure non-repudiation, special mechanisms are required such as digital signatures and receipts of mail. For institutions and companies, non-repudiation is especially essential to authority and responsibility. Company management personnel, such as supervisors, executives, and managers, need to manage or inspect different departments from time to time. Without proper authentication mechanisms, it is impossible for departments to verify the legitimacy of management personnel.

A company may be composed of multiple departments. When the company produces products by itself, it needs a factory. To reduce costs, the factory is usually located far away from the city or on the city’s outskirts. Due to the remote location, accessing networks may be difficult. Actually, plenty of authentication schemes need networks involved to transmit information for authentication. Some offline authentication schemes [20,21,22] were proposed to meet the requirements of specific applications.

Furthermore, online authentication schemes may not work under some specific situations. Firstly, even when networks are available, it is still hard to access them in some places such as the basement. Secondly, the failure of networks or the backend authentication server comes into play. Moreover, online authentication schemes need to transmit data to the remote authentication server. This approach raises the threat of various attacks.

Taking the above into consideration, to allow an executive to be authenticated by departments anonymously and offline while non-repudiation is ensured, we design an offline non-repudiation and anonymity-ensured user authentication system and propose an authentication scheme. In the system, every authorized executive will use his/her authentication device to help himself/herself to be authenticated by the department. To ensure anonymity, the department cannot authenticate the executive directly. Alternatively, the department will be issued a department authentication device. The department authentication device works as TPM (Trusted Platform Module). That is, it will tell the department whether the executive is legal instead of who the executive is. Meanwhile, who the executive is can be checked by the department authentication device such that non-repudiation can be ensured. If the executive is successfully authenticated by the department authentication device, he/she can deal with specific affairs. In the designed scheme, data will be transmitted between the executive’s authentication device and department’s authentication device. To protect anonymity, no one can reveal who the executive is by the transmitted data.

On the other hand, management personnel may change in the real world. For example, executive *A* who was originally assigned to manage department *a* is assigned to manage department *b*. In such a case, executive *A* is still a legal executive, and only his/her assignment changes. If any change of management personnel results in a significant change in all involved executives’ authentication data, this is cumbersome and annoying. In our designed user authentication scheme, authentication data stored in an executive’s authentication device does not need to be updated even when management personnel changes are made. To sum up, the proposed offline non-repudiation and anonymity-ensured authentication system needs to possess the following properties to comply with the desired requirements:

1. The legitimacy of a user can be verified offline.
2. Data transmitted between the department’s authentication device and the executive’s or system administrator’s authentication device must be protected.
3. Anonymity must be ensured.
4. Non-repudiation must be ensured.
5. Management can be done easily because authentication data stored in the executive’s authentication device does not need to be updated even when personnel changes are made.

The security of the proposed user authentication scheme is based on the factorization problem and discrete logarithm problem. In the designed system, a smartphone can be utilized as the executive’s authentication device and system administrator’s authentication device. This allows each party to store his/her personal authentication data privately. Although the proposed user authentication scheme is designed to help an executive be authenticated by the department authentication device, it can also be utilized for access control of small-sized enterprises/facilities/apartment complexes where workers/members/residents instead of executives are authenticated.

In the following, three examples are given to show what applications can adopt our proposed system.

**Example** **1.** *Alice is the company’s owner. Because there is only sufficient space to accommodate production demand in remote areas, factories of Alice’s company are located in remote areas. However, the network infrastructure in remote areas cannot comply with the requirements of real-time applications due to the limited transmission speed. That results in infeasible transmission delays and timeout events when authentication is proceeding. Moreover, employees in Alice’s factories may not know who the owner of factories is. Therefore, our proposed system can be utilized to have Alice successfully authenticated by her employees without the help of an authentication server and eliminate latent management threats.*

**Example** **2.** *Alice found out that the production output was less than ideal. She suspected that her employees did not use the manufacturing equipment properly, so she decided to personally inspect the job performance of her employees and equipment usage. Because she wants to inspect incognito, she can utilize our proposed system to allow these employees to ensure that Alice is authorized while her employees are unaware of who the inspector is.*

**Example** **3.** *Alice is the company’s owner and authorizes Eve to be an executive of factories. Unfortunately, Alice finds out that Eve does not work appropriately such that serious problems occur. Therefore, Alice wants to replace Eve with Bob. With our proposed system, Alice can easily revoke and delegate authorization.*

The rest of this paper is organized as follows. Preliminaries are introduced in Section 2. The architecture of the designed system is given in Section 3. The proposed user authentication scheme is shown in Section 4. Section 5 shows our property analysis and further security analysis, and demonstrates that the proposed scheme meets the requirements. The performance evaluation and further discussion are shown in Section 6. Finally, some conclusions are drawn in Section 7.

## 2. Preliminaries

To meet the specific properties of the proposed user authentication system, we designed the corresponding user authentication scheme whose security is based on the difficulties of solving the factoring problem and discrete logarithm problem. Two representative public-key cryptosystems, the RSA cryptosystem [23] and ElGamal cryptosystem [24], whose security are based on the difficulties of solving the factoring problem and discrete logarithm problem, respectively, are introduced.

### 2.1. RSA Cryptosystem

The RSA cryptosystem [23] proposed in 1978 was the first public-key cryptosystem. The security of the RSA cryptosystem is based on the difficulty of solving the factoring problem. The RSA cryptosystem possesses functions, encryption and decryption, and it can be used to generate a digital signature. The details are as follows:

Suppose there is a user *U*_1_. *U*_1_ must do the following to initialize the system.

Step 1: Choose two different large prime numbers, *p* and *q*, and compute *n* = *p* × *q.*
Step 2: Choose an integer *e* such that 1 < *e* < *ϕ*(*n*) and gcd(*e*, *ϕ*(*n*)) = 1, where *ϕ*(*n*) = (*p* − 1) (*q* − 1).
Step 3: Determine *d* such that *e* × *d* ≡ 1 (mod *ϕ*(*n*)).
Step 4: Keep *d* as his/her private key and make the corresponding public key (*e*, *n*) public.

When another user *U*_2_ wants to send a message *m* to *U*_1_ securely and only *U*_1_ can get *m*, *U*_2_ computes the ciphertext *C = m^e^* mod *n* with *U*_1_’s public key (*e*, *n*) for encryption and sends *C* to *U*_1_. After receiving *C*, *U*_1_ uses his/her private key *d* to compute *m = C^d^* mod *n* for decryption.

On the other hand, when *U*_1_ wants to generate a digital signature *S* for a message *m*, *U*_1_ uses his/her private key *d* to compute *S = m^d^* mod *n*. Then, when another user *U*_2_ gets *m* and *S* and wants to verify the digital signature, *U*_2_ uses *U*_1_’s public key (*e*, *n*) to compute *m*′ = *S^e^* mod *n* and checks if *m*′ and *m* are equal. If it holds, the signature *S* for *m* is verified, and *U*_2_ believes that *S* is generated by *U*_1_.

### 2.2. ElGamal Cryptosystem

The ElGamal cryptosystem [24] proposed in 1985 is another representative public-key cryptosystem, and its security is based on the difficulty of solving the discrete logarithm problem. The ElGamal cryptosystem possesses functions, encryption and decryption, and it can be used to generate a digital signature. To initialize the system, the following will be executed at first.

Step 1: A large prime number *p* and a generator *g* of GF(*p*) are chosen.
Step 2: For each user, an integer *x* in [1, *p* − 1] is chosen as the user’s private key, and the corresponding public key *y* is computed, where *y* = *g^x^* mod *p*.

Suppose there is a user *U*_1_, where *U*_1_’s private key is *x* and *U*_1_’s public key is *y* = *g^x^* mod *p*. Then, when another user *U*_2_ wants to send a message *m* to *U*_1_ securely, and only *U*_1_ can get *m*, *U*_2_ needs to execute the following steps.

Step 1: Choose a random number *r* in [1, *p* − 1].
Step 2: Compute *b* = *g^r^* mod *p* and *c* = *m* × *y^r^* mod *p*.
Step 3: Send the ciphertext (*b*, *c*) to *U*_1_.

After *U*_1_ receives the ciphertext (*b*, *c*), *U*_1_ computes *m* = *c* × (*b^x^*)^−1^ mod *p* for decryption.

On the other hand, when *U*_1_ wants to generate a digital signature *S* for a message *m*, *U*_1_ chooses a random number *k* in [1, *p* − 2] such that gcd(*k*, *p* − 1) = 1. Then, *U*_1_ computes *r* = *g^k^* mod *p* and *s*, where *m* = (*xr* + *ks*) mod (*p* − 1). (*r*, *s*) is the digital signature of *m.* When another user *U*_2_ gets *m* and (*r*, *s*) and wants to verify the digital signature, *U*_2_ checks whether *g^m^* mod *p* and *y*^r^*r*^s^ mod *p* are equal or not. If they are equal, the signature (*r*, *s*) for *m* is successfully verified, and *U*_2_ believes that (*r*, *s*) is generated by *U*_1_.

## 3. The Architecture of the Designed Offline Non-Repudiation and Anonymity-Ensured User Authentication System

**Figure 1:**
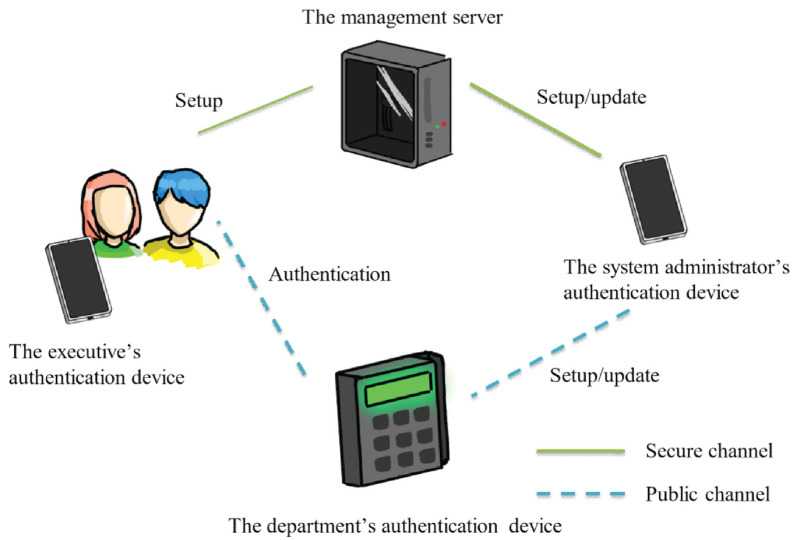
The architecture of the designed offline non-repudiation and anonymity-ensured user authentication system.

In the designed offline non-repudiation and anonymity-ensured user authentication system, there exist four entities: management server, system administrator’s authentication device, executive’s authentication device, and department’s authentication device, as shown in Figure 1. There is only one management server and only one system administrator in the system, and the system administrator is equipped with a system administrator’s authentication device. The numbers of executives and departments depend on the actual requirements. Each executive is equipped with an executive’s authentication device, and each department is equipped with an authentication device, namely a department authentication device. The detailed functions of these four entities are shown in the following.

1. Functions of the management server
  (a) Manage all authorization information, including information about various departments and the related authorized management personnel.
  (b) Generate all required parameters for authorization and authentication.
  (c) Store the authentication data in the system administrator’s authentication device and executive’s authentication device through a secure channel when the system is initialized.
  (d) Generate new authentication data and store it in the system administrator’s authentication device through a secure channel because of the change of authorized management personnel.
2. Functions of the system administrator’s authentication device
  (a) Set the authentication parameters on the department’s authentication device through a public channel.
  (b) Update the authentication parameters on the department’s authentication device through a public channel when authorized management personnel of this department changes.
3. The executive’s authentication device
  (a) Generate a nonce and send the authentication request to the department’s authentication device for authentication.
  (b) Verify the legitimacy of the department’s authentication device by the response of the department’s authentication device.
  (c) Compute the authentication parameters for the department’s authentication device to allow the department’s authentication device to verify the legitimacy and ensure non-repudiation of the executive.
4. The department’s authentication device
  (a) Verify the legitimacy of the system administrator’s authentication device before the stored authentication data is set or updated.
  (b) Generate a nonce and send it back to the executive’s authentication device after getting the authentication request from the administrator’s authentication device.
  (c) Verify the legitimacy and ensure non-repudiation of the executive after receiving the authentication parameters generated by the executive’s authentication device.

## 4. The User Authentication Scheme for the Designed Offline Non-Repudiation and Anonymity-Ensured User Authentication System

To ensure that the designed offline non-repudiation and anonymity-ensured user authentication system can meet the specific requirements, the proposed user authentication scheme needs to comply with the following:

1. The legitimacy of a user can be verified offline.
2. Data transmitted between the department’s authentication device and the executive’s or system administrator’s authentication device must be protected.
3. Anonymity must be ensured.
4. Non-repudiation must be ensured.
5. Management can be easily conducted because authentication data stored in the executive’s authentication device does not need to be updated even when personnel changes are made.

The proposed user authentication scheme is composed of four phases: initialization phase, department authentication device setup phase, authentication phase, and authentication data update phase. The notations used in the designed authentication scheme are defined in Table 1.

### 4.1. Initialization Phase

In the initialization phase, the management server *Server* first determines *Set*_1_ and *Set*_2_. Then, *Server* initializes a department *C_j_*’s department authentication device *D_j_*. *Server* computes *G_j_* = *H*(${\mathrm{ID}}_{D_{j}}$||*Master*_2_) and stores {*H*(.), *n*, *g*, *MS*, ${\mathrm{ID}}_{D_{j}}$, *G_j_*} in *D_j_*. Then, *C_j_* is equipped with *D_j_*.

**Figure 2:**
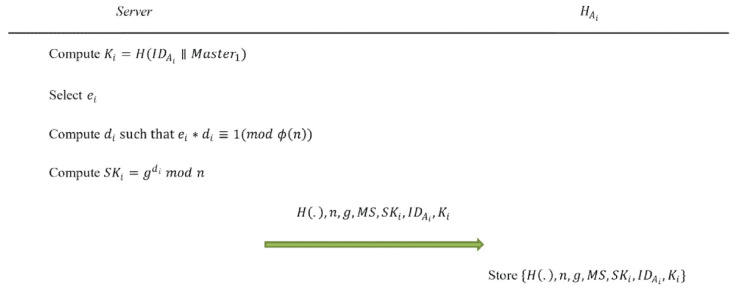
The process of the management server to initialize the executive’s authentication device in the initialization phase of the proposed user authentication scheme.

**Figure 3:**
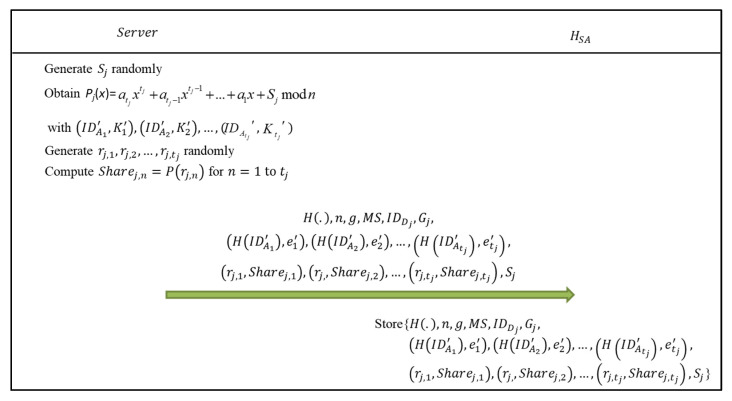
The process of the management server to initialize the system administrator’s authentication device in the initialization phase of the proposed user authentication scheme.

After confirming the corresponding executives of all departments, *Server* computes the authentication data for all executives of *Set*_1_ and departments of *Set*_2_, stores personal authentication data of the executive *A_i_* in his/her authentication device $H_{A_{i}}$, and stores department authentication data in *SA*’s authentication device *H_SA_*. In the initialization phase, data is transmitted through a secure channel. The initialization phase is depicted in Figure 2 and Figure 3, and the details are as follows:

Step 1: The management server *Server* computes *K_i_* = *H*(${\mathrm{ID}}_{A_{i}}$||*Master*_1_) for the executive *A_i_*, where *I* = 1, 2, …, *m.*
Step 2: *Server* selects *e_i_* for *A_i_* and then computes *d_i_* such that *d_i_* × *e_i_* ≡ 1 (mod *ϕ*(*n*)), where gcd(*e_i_*, *ϕ*(*n*)) = 1 and *e_r_* ≠ *e_α_* when *r* ≠ *α*.
Step 3: *Server* computes *SK_i_* = $g^{d_{i}}$ mod *n* for *A_i_*.
Step 4: *Server* stores *H*(.), *n*, *g*, *MS*, *SK*_*i*_,${\;\mathrm{ID}}_{A_{i}}$ and *K_i_* in *A_i_*’s authentication device $H_{A_{i}}$.
Step 5: *Server* randomly generates a dedicated authentication code *S_j_* for *C_j_*’s department authentication device *D_j_*, where *S_j_* < *n* and *j* = 1, 2, …, *w*.
Step 6: *Server* uses (${{\mathrm{ID}}_{A_{1}}}^{\prime }$, *K*_1_′), (${{\mathrm{ID}}_{A_{2}}}^{\prime }$, *K*_2_′), …, (${{\mathrm{ID}}_{A_{t_{j}}}}^{\prime }$, ${K_{t_{j}}}^{\prime }$) of executives *A*_1_′, *A*_2_′, …, ${A_{t_{j}}}^{\prime }$ who are authorized to manage or inspect *C_j_*, and (0, *S_j_*) to obtain the polynomial *P_j_*(*x*) = $a_{t_{j}}x^{t_{j}}\;$ + $\;a_{t_{j}-1}x^{t_{j}-1}$ + … + *a*_1_*x* + *S_j_* mod *n*, where *t_j_* is the number of authorized executives who can manage or inspect *C_j_*, {*A*_1_′, *A*_2_′, …, $A_{t_{j}}{}^{\prime }$}⊆{*A_i_*|*I* = 1, 2, …, *m*}, *P_j_*(0) = *S_j_*, *P_j_*(${\mathrm{ID}}_{A_{1}}{}^{\prime }$) = *K*_1_′, *P_j_*(${\mathrm{ID}}_{A_{2}}{}^{\prime }$) = *K*_2_′, …, and *P_j_*(${\mathrm{ID}}_{A_{t_{j}}}{}^{\prime }$) = $K_{t_{j}}{}^{\prime }$.
Step 7: *Server* randomly generates *r_j,_*_1_, *r_j,_*_2_, …, $r_{{j,t}_{j}}$ and computes *Share_j,_*_1_ = *P_j_*(*r_j,_*_1_), *Share_j,_*_2_ = *P_j_*(*r_j,_*_2_), …, ${\mathrm{Share}}_{{j,t}_{j}}$ = *P_j_*($r_{{j,t}_{j}}$), where *r_j,_*_1_, *r_j,_*_2_, …, $r_{{j,t}_{j}}$ are less than min(*p*, *q*), *r_j,_*_1_, *r_j,_*_2_, …, $r_{{j,t}_{j}}$ are different from each other and *r_j,_*_1_, *r_j,_*_2_, …, $r_{{j,t}_{j}}$ are different from ${\mathrm{ID}}_{A_{1}}{}^{\prime }$, ${\mathrm{ID}}_{A_{2}}{}^{\prime }$, …, ${\mathrm{ID}}_{A_{t_{j}}}{}^{\prime }$.
Step 8: *Server* stores *H*(.), *n*, *g*, *MS*, ${\mathrm{ID}}_{D_{j}}$, *G_j_*, (*H*(${\mathrm{ID}}_{A_{1}}{}^{\prime }$), *e*_1_′), (*H*(${\mathrm{ID}}_{A_{2}}{}^{\prime }$), *e*_2_′), …, (*H*(${\mathrm{ID}}_{A_{t_{j}}}{}^{\prime }$), $e_{t_{j}}{}^{\prime }$), (*r_j,_*_1_, *Share_j,_*_1_), (*r_j,_*_2_, *Share_j,_*_2_), …, ($r_{{j,t}_{j}}$, ${\mathrm{Share}}_{{j,t}_{j}}$) and *S_j_* in the system administrator *SA*’s authentication device *H_SA_*, where *j* = 1, 2, …, *w*.

### 4.2. Department Authentication Device Setup Phase

**Figure 4:**
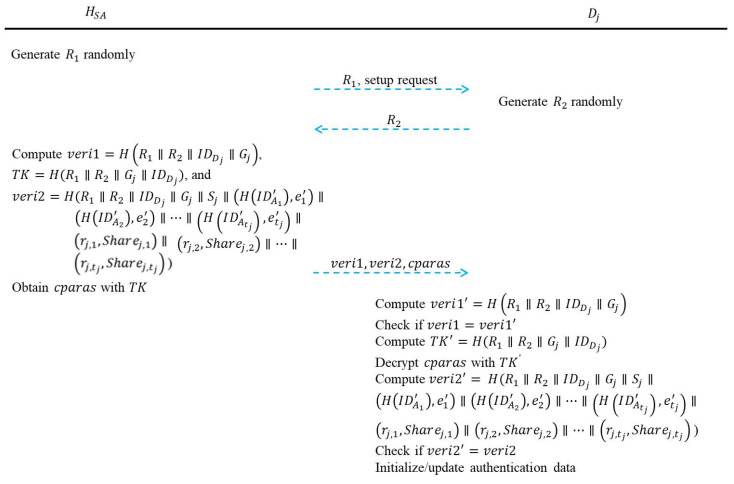
Department authentication device setup phase of the proposed user authentication scheme.

This phase will be executed when the system administrator *SA* wants to initialize or update the authentication data in *C_j_*’s department authentication device *D_j_*. Data is transmitted through a public channel in this phase. The department authentication device setup phase is depicted in Figure 4, and the details are as follows:

Step 1: *H_SA_* generates a random number *R*_1_ and sends *R*_1_ with a setup request to *D_j_*.
Step 2: When *D_j_* receives *R*_1_ and a setup request from *H_SA_*, *D_j_* generates a random number *R*_2_. Then, *D_j_* sends *R*_2_ back to *H_SA_*.
Step 3: When *H_SA_* receives *R*_2_, *H_SA_* computes *veri*1 = *H*(*R*_1_||*R*_2_||${\mathrm{ID}}_{D_{j}}$||*G_j_*), *TK* = *H*(*R*_1_||*R*_2_||*G_j_*||${\mathrm{ID}}_{D_{j}}$) and *veri*2 = *H*(*R*_1_||*R*_2_||${\mathrm{ID}}_{D_{j}}$||*G_j_*||*S_j_*||(*H*(${\mathrm{ID}}_{A_{1}}{}^{\prime }$), *e*_1_′)||(*H*(${\mathrm{ID}}_{A_{2}}{}^{\prime }$), *e*_2_′)||…||(*H*(${\mathrm{ID}}_{A_{t_{j}}}{}^{\prime }$), $e_{t_{j}}{}^{\prime }$)||(*r_j,_*_1_, *Share_j,_*_1_)||(*r_j,_*_2_, *Share_j,_*_2_)||…||($r_{{j,t}_{j}}$, ${\mathrm{Share}}_{{j,t}_{j}}$). Additionally, *H_SA_* encrypts {*S_j_*, (*H*(${\mathrm{ID}}_{A_{1}}{}^{\prime }$), *e*_1_′), (*H*(${\mathrm{ID}}_{A_{2}}{}^{\prime }$), *e*_2_′), …, (*H*(${\mathrm{ID}}_{A_{t_{j}}}{}^{\prime }$), $e_{t_{j}}{}^{\prime }$), (*r_j,_*_1_, *Share_j,_*_1_), (*r_j,_*_2_, *Share_j,_*_2_), …, ($r_{{j,t}_{j}}$, ${\mathrm{Share}}_{{j,t}_{j}}$)} with *TK* to get *cparas*.
Step 4: *H_SA_* sends *veri*1, *veri*2, and *cparas* to *D_j_*.
Step 5: After receiving *veri*1, *veri*2, and *cparas*, *D_j_* first uses *ID_D_j__* and *G_j_* to compute *veri*1′ = *H*(*R*_1_||*R*_2_||${\mathrm{ID}}_{D_{j}}$||*G_j_*). If *veri*1′ is equal to *veri*1, *D_j_* computes *TK*′ = *H*(*R*_1_||*R*_2_||*G_j_*||${\mathrm{ID}}_{D_{j}}$), decrypts *cparas* with *TK*′ to obtain {*S_j_*, (*H*(${\mathrm{ID}}_{A_{1}}{}^{\prime }$), *e*_1_′), (*H*(${\mathrm{ID}}_{A_{2}}{}^{\prime }$), *e*_2_′), …, (*H*(${\mathrm{ID}}_{A_{t_{j}}}{}^{\prime }$), $e_{t_{j}}{}^{\prime }$), (*r_j,_*_1_, *Share_j,_*_1_), (*r_j,_*_2_, *Share_j,_*_2_), …, ($r_{{j,t}_{j}}$, ${\mathrm{Share}}_{{j,t}_{j}}$)}, and computes *veri*2′ = *H*(*R*_1_||*R*_2_||${\mathrm{ID}}_{D_{j}}$||*G_j_*||*S_j_*||(*H*(${\mathrm{ID}}_{A_{1}}{}^{\prime }$), *e*_1_′)||(*H*(${\mathrm{ID}}_{A_{2}}{}^{\prime }$), *e*_2_′)||…||(*H*(${\mathrm{ID}}_{A_{t_{j}}}{}^{\prime }$), $e_{t_{j}}{}^{\prime }$)||(*r_j,_*_1_, *Share_j,_*_1_)||(*r_j,_*_2_, *Share_j,_*_2_)||…||($r_{{j,t}_{j}}$, ${\mathrm{Share}}_{{j,t}_{j}}$)).
Step 6: If *veri*2′ is equal to *veri*2, the authentication data of *D_j_* is initialized or updated with *S_j_*, (*H*(${\mathrm{ID}}_{A_{1}}{}^{\prime }$), *e*_1_′), (*H*(${\mathrm{ID}}_{A_{2}}{}^{\prime }$), *e*_2_′), …, (*H*(${\mathrm{ID}}_{A_{t_{j}}}{}^{\prime }$), $e_{t_{j}}{}^{\prime }$), (*r_j,_*_1_, *Share_j,_*_1_), (*r_j,_*_2_, *Share_j,_*_2_), …, ($r_{{j,t}_{j}}$, ${\mathrm{Share}}_{{j,t}_{j}}$).

### 4.3. Authentication Phase

**Figure 5:**
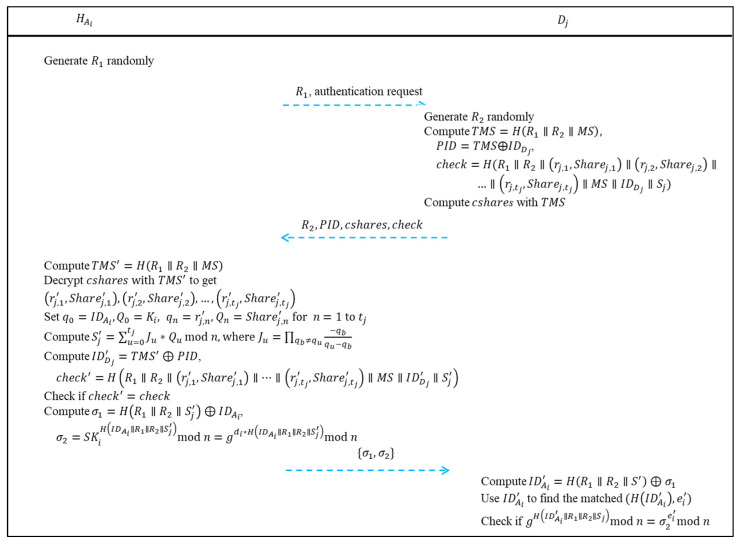
Authentication phase of the proposed user authentication scheme.

When the executive *A_i_* wants to deal with the management of *C_j_* or inspect *C_j_*, he/she needs to be authenticated by *C_j_*’s department authentication device *D_j_* with his/her authentication device $H_{A_{i}}$. In the authentication phase, *A_i_* can be authenticated by *D_j_* with the help of $H_{A_{i}}$ while *C_j_* cannot know who *A_i_* is, and data is transmitted through a public channel. The authentication phase is depicted in Figure 5, and the details are as follows:

Step 1: $H_{A_{i}}$ generates a random number *R*_1_ and sends *R*_1_ with an authentication request to *D_j_*.
Step 2: When *D_j_* receives *R*_1_ and the authentication request from $H_{A_{i}}$, *D_j_* generates a random number *R*_2_. Next, *D_j_* computes *TMS* = *H*(*R*_1_||*R*_2_||*MS*), *PID* = *TMS* ⊕ ${\mathrm{ID}}_{D_{j}}$ and *check* = *H*(*R*_1_||*R*_2_||(*r_j,_*_1_, *Share_j,_*_1_)||(*r_j,_*_2_, *Share_j,_*_2_)||…||($r_{{j,t}_{j}}$, ${\mathrm{Share}}_{{j,t}_{j}}$)||*MS*||${\mathrm{ID}}_{D_{j}}$||*S_j_*).
Step 3: *D_j_* encrypts (*r_j,_*_1_, *Share_j,_*_1_), (*r_j,_*_2_, *Share_j,_*_2_), …, ($r_{{j,t}_{j}}$, ${\mathrm{Share}}_{{j,t}_{j}}$) with *TMS* to get *cshares*.
Step 4: *D_j_* sends *R*_2_, *PID*, *cshares,* and *check* to $H_{A_{i}}$.
Step 5: After receiving *R*_2_, *PID*, *cshares* and *check*, $H_{A_{i}}$ computes *TMS*′ = *H*(*R*_1_||*R*_2_||*MS*) and then decrypts *cshares* with *TMS*′ to obtain (*r_j,_*_1_′, *Share_j,_*_1_′), (*r_j,_*_2_′, *Share_j,_*_2_′), …, ($r_{{j,t}_{j}}$′, ${\mathrm{Share}}_{{j,t}_{j}}$′).
Step 6: $H_{A_{i}}$ uses (*r_j,_*_1_′, *Share_j,_*_1_′), (*r_j,_*_2_′, *Share_j,_*_2_′), …, ($r_{{j,t}_{j}}$′, ${\mathrm{Share}}_{{j,t}_{j}}$′) and (${\mathrm{ID}}_{A_{i}}$, *K_i_*) to set the parameters *q*_0_ = ${\mathrm{ID}}_{A_{i}}$, *Q*_0_ = *K_i_*, *q*_1_ = *r_j,_*_1_′, *Q*_1_ = *Share_j,_*_1_′, *q*_2_ = *r_j,_*_2_′, *Q*_2_ = *Share_j,_*_2_′, …, $q_{t_{j}}$ = $r_{{j,t}_{j}}$′, $Q_{t_{j}}$ = ${\mathrm{Share}}_{{j,t}_{j}}$′. Then, $H_{A_{i}}$ computes *S_j_*′ = $\sum_{u=0}^{t_{j}}J_{u}{\times Q}_{u}$ mod *n*, where $J_{u}=\prod_{q_{b}\neq q_{u}}\frac{{-q}_{b}}{q_{u}{-q}_{b}}$.
Step 7: $H_{A_{i}}$ computes ${\mathrm{ID}}_{D_{j}}{}^{\prime }$ = *TMS*′ ⊕ *PID* and *check*′ = *H*(*R*_1_||*R*_2_||(*r_j,_*_1_′, *Share_j,_*_1_′)||(*r_j,_*_2_′, *Share_j,_*_2_′)||…||($r_{{j,t}_{j}}$′, ${\mathrm{Share}}_{{j,t}_{j}}$′)||*MS*||${\mathrm{ID}}_{D_{j}}$′||*S_j_*′) and checks whether *check*′ and *check* are equal or not. If it holds, it denotes that *D_j_* is indeed a legal department authentication device, *D_j_*’s identity is indeed ${\mathrm{ID}}_{D_{j}}$, and the derived *S_j_*′ is correct.
Step 8: $H_{A_{i}}$ computes *σ*_1_* = H*(*R*_1_||*R*_2_||*S_j_*′) ⊕ ${\mathrm{ID}}_{A_{i}}$ and *σ*_2_ = ${\mathrm{SK}}_{i}^{{H(\mathrm{ID}}_{A_{i}}{||R}_{1}{||R}_{2}{||S}_{j}{}^{\prime })}$ mod *n* = $g^{d_{i}{\times H(\mathrm{ID}}_{A_{i}}{||R}_{1}{||R}_{2}{||S}_{j}{}^{\prime })}$ mod *n*. Then, $H_{A_{i}}$ sends *σ*_1_ and *σ*_2_ to *D_j_*.
Step 9: After receiving *σ*_1_ and *σ*_2_, *D_j_* computes ${\mathrm{ID}}_{A_{i}}{}^{\prime }$ = *H*(*R*_1_||*R*_2_||*S_j_*) ⊕ *σ*_1_. Then, *D_j_* uses ${\mathrm{ID}}_{A_{i}}{}^{\prime }$ as the index to find the matched (*H*(${\mathrm{ID}}_{A_{i}}{}^{\prime }$), *e_i_*′) in (*H*(${\mathrm{ID}}_{A_{1}}{}^{\prime }$), *e*_1_′), (*H*(${\mathrm{ID}}_{A_{2}}{}^{\prime }$), *e*_2_′), …, (*H*(${\mathrm{ID}}_{A_{t_{j}}}{}^{\prime }$), $e_{t_{j}}{}^{\prime }$).
Step 10: *D_j_* checks whether $g^{{H(\mathrm{ID}}_{A_{i}}{{}^{\prime }||R}_{1}{||R}_{2}{||S}_{j})}$ mod *n* and $\sigma_{2}^{e_{i}{}^{\prime }}$ mod *n* are equal or not. If they are equal, it denotes that *A_i_* is a legal executive, and *A_i_*’s identity is ${\mathrm{ID}}_{A_{i}}{}^{\prime }$. That is, *A_i_* is authenticated by *D_j_* with $H_{A_{i}}$’s help, and he/she can then deal with the management of *C_j_* or inspect *C_j_*.

### 4.4. Authentication Data Update Phase

When the authorized management personnel of a department changes, the authentication data update phase will be executed. If a new executive joins, this phase will be executed from Step 1. If the changes do not result in the joining of a new executive, this phase will be executed from Step 5. The management server *Server* computes the updated authentication data for the authentication devices of all departments that are influenced by the changes. Thereupon, the system administrator *SA* uses his/her authentication device *H_SA_* to update the authentication data stored in the corresponding department’s authentication device. The steps are shown as follows:

Step 1: *Server* computes *K_i_*′ = *H*(${\mathrm{ID}}_{A_{1}{}^{\prime }}$||*Master*_1_) for the new executive *A_i_*′.
Step 2: *Server* selects *e_i_*′ for *A_i_*′, and computes *d_i_*′ such that *d_i_*′× *e_i_*′ ≡ 1 (mod *ϕ*(*n*)), where gcd(*e_i_*′, *ϕ*(*n*)) = 1 and *e_i_*′ is different from the existing *e_i_*’s.
Step 3: *Server* computes *SK_i_*′ = ${\;g}^{d_{i}{}^{\prime }}$ mod *n* for *A_i_*′.
Step 4: *Server* stores *H*(.), *n*, *g*, *MS*, *SK_i_*′, ${\mathrm{ID}}_{A_{1}{}^{\prime }}$ and *K_i_*′ in the authentication device $H_{A_{1}{}^{\prime }}$ of the new executive *A_i_*′.
Step 5: *Server* randomly generates a dedicated authentication code *S_j_*′ for the department authentication device *D_j_*′ of the influenced department *C_j_*′, where *D_j_*′∈{*D_k_*|*k* = 1, 2, …, *w*}.
Step 6: *Server* uses (${\mathrm{ID}}_{A_{1}}{}^{\prime }$, *K*_1_′), (${\mathrm{ID}}_{A_{2}}{}^{\prime }$, *K*_2_′), …, (${\mathrm{ID}}_{A_{t_{j}}}{}^{\prime }$, $K_{t_{j}}{}^{\prime }$) of management personnel *A*_1_′, *A*_2_′, …, $A_{t_{j}}{}^{\prime }$ who can manage or inspect *C_j_*′ and (0, *S_j_*′) to obtain the polynomial *P_j_*′(*x*) = $a_{t_{j}{}^{\prime }}x^{t_{j{}^{\prime }}}$ + $a_{t_{j}{}^{\prime }-1}x^{t_{j}{}^{\prime }-1}$ + …+ *a*_1_*x* + *S_j_*′ mod *n*, where *t_j_*′ is the number of management personnel who can manage or inspect *C_j_*′, {*A*_1_′, *A*_2_′, …, $A_{t_{j}}{}^{\prime }$} ⊆ {*A_i_*|*i* = 1, 2, …, *m*}, subject to *P_j_*′(${\mathrm{ID}}_{A_{1}}{}^{\prime }$) = *K*_1_′, *P_j_*′(${\mathrm{ID}}_{A_{2}}{}^{\prime }$) = *K*_2_′, …, and *P_j_*′(${\mathrm{ID}}_{A_{t_{j}}}{}^{\prime }$) = $\;K_{t_{j}}{}^{\prime }$.
Step 7: *Server* randomly generates *r_j,_*_1_, *r_j,_*_2_, …, $r_{{j,t}_{j}{}^{\prime }}$ and computes *Share _j,_*_1_ = *P_j_*′(*r_j,_*_1_), *Share _j,_*_2_ = *P_j_*′(*r_j,_*_2_), …, ${\mathrm{Share}}_{{j,t}_{j}}$ = *P_j_*′($r_{{j,t}_{j}{}^{\prime }}$), where *r*_*j*,__1_, *r*_*j*,__2_, …, $r_{{j,t}_{j}{}^{\prime }}$ are less than min(*p*, *q*), *r_j,_*_1_, *r_j,_*_2_, …, $r_{{j,t}_{j}{}^{\prime }}$ are different from each other and *r_j,_*_1_, *r_j,_*_2_, …, $r_{{j,t}_{j}{}^{\prime }}$ are different from ${\mathrm{ID}}_{A_{1}}{}^{\prime }$, ${\mathrm{ID}}_{A_{2}}{}^{\prime }$, …, ${\mathrm{ID}}_{A_{t_{j}}}{}^{\prime }$.
Step 8: *Server* stores ${\mathrm{ID}}_{D_{j}{}^{\prime }}$, (*H*(${\mathrm{ID}}_{A_{1}}{}^{\prime }$), *e*_1_′), (*H*(${\mathrm{ID}}_{A_{2}}{}^{\prime }$), *e*_2_′), …, (*H*(${\mathrm{ID}}_{A_{t_{j}}{}^{\prime }}{}^{\prime }$), $e_{t_{j}{}^{\prime }}{}^{\prime }$), (*r_j,_*_1_′, *Share_j,_*_1_′), (*r_j,_*_2_′, *Share_j,_*_2_′), …, ($r_{{j,t}_{j}{}^{\prime }}$′, ${\mathrm{Share}}_{{j,t}_{j}{}^{\prime }}$′) and *S_j_*′ in *SA*’s authentication device *H_SA_*.
Step 9: *H_SA_* executes the department authentication device setup phase to update the authentication data stored in the influenced department *C_j_*′’s authentication device *D_j_*′.

## 5. Property Analysis and Further Analysis

In the following, property analysis is first made to demonstrate that the five properties secretly mentioned are ensured to meet the requirements of the designed offline non-repudiation and anonymity-ensured authentication system. Then, comparisons between authentication schemes ensuring anonymity and ours are made. Finally, further security analysis is conducted to show that our scheme can resist common attacks and the correctness is ensured.

### 5.1. Property Analysis

As previously mentioned, the proposed offline non-repudiation and anonymity-ensured authentication system needs to possess the following properties to comply with the desired requirements:

1. The legitimacy of a user can be verified offline.
2. Data transmitted between the department’s authentication device and the executive’s or system administrator’s authentication device must be protected.
3. Anonymity must be ensured.
4. Non-repudiation must be ensured.
5. Management can be easily conducted because authentication data stored in the executive’s authentication device does not need to be updated even when personnel changes are made.

The corresponding analysis is performed as follows.

#### 5.1.1. Offline Authentication

In the authentication phase, if executive *A_i_* wants to manage or inspect *C_j_*, he/she must be authenticated by the authentication device *D_j_* of *C_j_* with his/her own authentication device $H_{A_{i}}$. *A_i_* can authenticate *D_j_* independently without the management server *Server*’s help, and *A_i_* authenticates *D_j_* by checking whether *check*′ and *check* are equal or not, where *check* = *H*(*R*_1_||*R*_2_||(*r_j,_*_1_, *Share_j,_*_1_)||(*r_j,_*_2_, *Share_j,_*_2_)||…||($r_{{j,t}_{j}}$, ${\mathrm{Share}}_{{j,t}_{j}}$)||*MS*||${\mathrm{ID}}_{D_{j}}$||*S_j_*). Meanwhile, *D_j_* also verifies the legitimacy of *A_i_* by checking whether $g^{{H(\mathrm{ID}}_{A_{i}}{}^{\prime }{||R}_{1}{||R}_{2}{||S}_{j})}$ mod *n* and $\sigma_{2}^{e_{i}{}^{\prime }}$ mod *n* are equal or not. That is, the legitimacy of a user can be verified offline.

#### 5.1.2. Protection of the Transmitted Data

Assume that adversary 𝒜 intercepts all messages transmitted in the department authentication device setup phase and authentication phase. *H_SA_* sends *veri*1, *veri*2, and *cparas* to *D_j_* in the department authentication device setup phase, where *veri*1 = *H*(*R*_1_||*R*_2_||${\mathrm{ID}}_{D_{j}}$||*G_j_*), *veri*2 = *H*(*R*_1_||*R*_2_||${\mathrm{ID}}_{D_{j}}$||*G_j_*||*S_j_*||(*H*(${\mathrm{ID}}_{A_{1}}{}^{\prime }$), *e*_1_′)||(*H*(${\mathrm{ID}}_{A_{2}}{}^{\prime }$), *e*_2_′)||…||(*H*(${\mathrm{ID}}_{A_{t_{j}}}{}^{\prime }$), $e_{t_{j}}{}^{\prime }$)||(*r_j,_*_1_, *Share_j,_*_1_)||(*r_j,_*_2_, *Share_j,_*_2_)||…||($r_{{j,t}_{j}}$, ${\mathrm{Share}}_{{j,t}_{j}}$)), and *cparas* is the ciphertext of (*S_j_*, (*H*(${\mathrm{ID}}_{A_{1}}{}^{\prime }$), *e*_1_′), (*H*(${\mathrm{ID}}_{A_{2}}{}^{\prime }$), *e*_2_′), …, (*H*(${\mathrm{ID}}_{A_{t_{j}}}{}^{\prime }$), $e_{t_{j}}{}^{\prime }$), (*r_j,_*_1_, *Share_j,_*_1_), (*r_j,_*_2_, *Share_j,_*_2_), …, ($r_{{j,t}_{j}}$, ${\mathrm{Share}}_{{j,t}_{j}}$)). Because of the properties of one-way hash functions, it is hard for 𝒜 to retrieve the unknown parameters, such as ${\mathrm{ID}}_{D_{j}}$, *G_j_* and *S_j_*, from *veri*1 and *veri*2. On the other hand, *cparas* is the ciphertext of (*S_j_*, (*H*(${\mathrm{ID}}_{A_{1}}{}^{\prime }$), *e*_1_′), (*H*(${\mathrm{ID}}_{A_{2}}{}^{\prime }$), *e*_2_′), …, (*H*(${\mathrm{ID}}_{A_{t_{j}}}{}^{\prime }$), $e_{t_{j}}{}^{\prime }$), (*r_j,_*_1_, *Share_j,_*_1_), (*r_j,_*_2_, *Share_j,_*_2_), …, ($r_{{j,t}_{j}}$, ${\mathrm{Share}}_{{j,t}_{j}}$)) with the encryption key *TK* = *H*(*R*_1_||*R*_2_||*G_j_*||${\mathrm{ID}}_{D_{j}}$). Because *G_j_* and ${\mathrm{ID}}_{D_{j}}$ are unknown, 𝒜 cannot obtain *TK* to decrypt *cparas* to retrieve *S_j_*. On the other hand, in the authentication phase, *D_j_* sends *R*_2_, *PID*, *cshares*, and *check* to $H_{A_{i}}$, where *PID* = *TMS* ⊕ ${\mathrm{ID}}_{D_{j}}$, *check* = *H*(*R*_1_||*R*_2_||(*r_j,_*_1_, *Share_j,_*_1_)||(*r_j,_*_2_, *Share_j,_*_2_)||…||($r_{{j,t}_{j}}$, ${\mathrm{Share}}_{{j,t}_{j}}$)||*MS*||${\mathrm{ID}}_{D_{j}}$||*S_j_*), and *cshares* is the ciphertext of ((*r_j,_*_1_, *Share_j,_*_1_), (*r_j,_*_2_, *Share_j,_*_2_), …, ($r_{{j,t}_{j}}$, ${\mathrm{Share}}_{{j,t}_{j}}$)) with the encryption key *TMS = H*(*R*_1_||*R*_2_||*MS*). Then, $H_{A_{i}}$ sends *σ*_1_ and *σ*_2_, where *σ*_1_ = *H*(*R*_1_||*R*_2_||*S_j_*’) ⊕ ${\mathrm{ID}}_{A_{i}}$ and *σ*_2_ = ${\mathrm{SK}}_{i}^{{H(\mathrm{ID}}_{A_{i}}{||R}_{1}{||R}_{2}{||S}_{j}{}^{\prime })}$ mod *n*. Firstly, because ${\mathrm{ID}}_{D_{j}}$ is concealed and not transmitted, 𝒜 cannot retrieve *TMS* from *PID*. Secondly, because of the properties of one-way hash functions, it is hard for 𝒜 to retrieve the unknown parameters, such as *MS*, ${\mathrm{ID}}_{D_{j}}$ and *S_j_*, from *check*. Thirdly, because *MS* is unknown, 𝒜 cannot obtain *TMS* to decrypt *cshares* or retrieve ${\mathrm{ID}}_{D_{j}}$ from *PID*. Fourthly, because *S_j_* is unknown, it is impossible for 𝒜 to retrieve ${\mathrm{ID}}_{A_{i}}$ from *σ*_1_.

Because of the above, it is ensured that data transmitted between the department’s authentication device and the executive’s or system administrator’s authentication device is protected.

#### 5.1.3. Anonymity and Untraceability

In the proposed scheme, the identities of all entities are not transmitted without being concealed through public channels. Firstly, *D_j_* sends *PID* and *check* to $H_{A_{i}}$, where *PID* = *TMS* ⊕ ${\mathrm{ID}}_{D_{j}}$, *check* = *H*(*R*_1_||*R*_2_||(*r_j,_*_1_, *Share_j,_*_1_)||(*r_j,_*_2_, *Share_j,_*_2_)||…||($r_{{j,t}_{j}}$, ${\mathrm{Share}}_{{j,t}_{j}}$)||*MS*||${\mathrm{ID}}_{D_{j}}$||*S_j_*), and *TMS* = *H*(*R*_1_||*R*_2_||*MS*). Because of the properties of one-way hash functions, it is hard to retrieve the unknown parameters, such as *MS*, ${\mathrm{ID}}_{D_{j}}$ and *S_j_*, from *check*. Thus, *TMS* cannot be obtained to retrieve ${\mathrm{ID}}_{D_{j}}$ from *PID*. Then, $H_{A_{i}}$ sends *σ*_1_ and *σ*_2_, where *σ*_1_ = *H*(*R*_1_||*R*_2_||*S_j_*’) ⊕ ${\mathrm{ID}}_{A_{i}}$ and *σ*_2_ = ${\mathrm{SK}}_{i}^{{H(\mathrm{ID}}_{A_{i}}{||R}_{1}{||R}_{2}{||S}_{j}{}^{\prime })}$ mod *n*. Because *S_j_* is unknown, it is impossible to retrieve ${\mathrm{ID}}_{A_{i}}$ from *σ*_1_. Furthermore, all parameters transmitted in the authentication phase are computed with fresh random numbers *R*_1_ and *R*_2_. Consequently, transmitted parameters in one session must differ from those in other sessions.

According to the above, it is shown that no one can trace a specific entity or reveal the communication party’s identity. Thus, anonymity and untraceability are ensured in the proposed scheme.

#### 5.1.4. Non-Repudiation

In the authentication phase, *D_j_* sends *R*_2_, *PID*, *cshares*, and *check* to $H_{A_{i}}$, where *PID* = *TMS* ⊕ ${\mathrm{ID}}_{D_{j}}$, *check* = *H*(*R*_1_||*R*_2_||(*r_j,_*_1_, *Share_j,_*_1_)||(*r_j,_*_2_, *Share_j,_*_2_)||…||($r_{{j,t}_{j}}$, ${\mathrm{Share}}_{{j,t}_{j}}$)||*MS*||${\mathrm{ID}}_{D_{j}}$||*S_j_*), and *cshares* is the ciphertext of ((*r_j,_*_1_, *Share_j,_*_1_), (*r_j,_*_2_, *Share_j,_*_2_), …, ($r_{{j,t}_{j}}$, ${\mathrm{Share}}_{{j,t}_{j}}$)) with the encryption key *TMS* = *H*(*R*_1_||*R*_2_||*MS*). After receiving *R*_2_, *PID*, *cshares* and *check*, $H_{A_{i}}$ computes *TMS*′ = *H*(*R*_1_||*R*_2_||*MS*), decrypts *cshares* with *TMS*′ to retrieve (*r_j,_*_1_′, *Share_j,_*_1_′), (*r_j,_*_2_′, *Share_j,_*_2_′), …, ($r_{{j,t}_{j}}$′, ${\mathrm{Share}}_{{j,t}_{j}}$′), and uses these shares and (${\mathrm{ID}}_{A_{i}}$, *K_i_*) to obtain *S_j_*′. Then, $H_{A_{i}}$ computes ${\mathrm{ID}}_{D_{j}}$′ = *TMS*′ ⊕ *PID* and *check*′ = *H*(*R*_1_||*R*_2_||(*r_j,_*_1_′, *Share_j,_*_1_′)||(*r_j,_*_2_′, *Share_j,_*_2_′)||…||($r_{{j,t}_{j}}$′, ${\mathrm{Share}}_{{j,t}_{j}}$′)||*MS*||${\mathrm{ID}}_{D_{j}}$′||*S_j_*′) and checks whether *check*′ and *check* are equal or not. Because ${\mathrm{ID}}_{D_{j}}$ is concealed when it is transmitted and only *D_j_* knows both ${\mathrm{ID}}_{D_{j}}$ and *S_j_*, only *D_j_* can compute *check* to be successfully authenticated by $H_{A_{i}}$.

On the other hand, $H_{A_{i}}$ computes *σ*_1_ = *H*(*R*_1_||*R*_2_||*S_j_*’) ⊕ ${\mathrm{ID}}_{A_{i}}$ and *σ*_2_ = ${\mathrm{SK}}_{i}^{{H(\mathrm{ID}}_{A_{i}}{||R}_{1}{||R}_{2}{||S}_{j}{}^{\prime })}$ mod *n*. Then, $H_{A_{i}}$ send *σ*_1_ and *σ*_2_ to *D_j_*. After receiving *σ*_1_ and *σ*_2_, *D_j_* computes ${\mathrm{ID}}_{A_{i}}{}^{\prime }$ = *H*(*R*_1_||*R*_2_||*S_j_*) ⊕ *σ*_1_. Then, *D_j_* uses ${\mathrm{ID}}_{A_{i}}{}^{\prime }$ as the index to find the matched (*H*(${\mathrm{ID}}_{A_{i}}{}^{\prime }$), *e_i_*′) and checks whether $g^{{H(\mathrm{ID}}_{A_{i}}{}^{\prime }{||R}_{1}{||R}_{2}{||S}_{j})}$ mod *n* and $\sigma_{2}^{e_{i}{}^{\prime }}$ mod *n* are equal or not. Because ${\mathrm{ID}}_{A_{i}}$ is concealed when it is transmitted and only $H_{A_{i}}$ knows both ${\mathrm{ID}}_{A_{i}}$ and *SK_i_*, only $H_{A_{i}}$ can compute *σ*_2_ to be successfully authenticated by *D_j_*. Consequently, the proposed scheme ensures non-repudiation.

#### 5.1.5. Simplified Management

When the authorized management personnel of a department changes, the authentication data update phase will be executed. If a new executive joins, *Server* only computes *K_i_*′, *d_i_*′, and *SK_i_*′ for the new executive *A_i_*′ and stores the parameters in *A_i_*’s $H_{A_{i}{}^{\prime }}$. Then, *Server* only needs to compute the required parameters for the influenced departments while authentication data kept by the remaining executives does not need to be updated. This approach can greatly eliminate extra burdens and simplify management.

### 5.2. Comparisons between Authentication Schemes Ensuring Anonymity and the Proposed User Authentication Scheme

The proposed user authentication scheme ensures anonymity. To show that our scheme possesses superior properties, comparisons between authentication schemes ensuring anonymity [5,6,11,12,13,14,15,16,17,18,19] and the proposed user authentication scheme are made as follows. Authentication schemes [5,15,16,17] proposed for healthcare use biometrics as a factor to authenticate users, and this approach produces extra components to extract the biometrics needed. Authentication schemes were proposed for IoT applications [6,11,12], VANET [13,18,19], and cloud computing applications [13,14]. Users in these authentication schemes [5,6,11,12,13,14,15,16,17,18,19] need to register with a trusted authentication server, and be authenticated online when accessing services, where users may be authenticated by the trusted authentication server directly or by other servers with the trusted authentication server’s help. In the proposed scheme, a user/executive can be authenticated offline, and no extra component is needed. These properties enable the proposed scheme to work well without being influenced by the failure of networks or the backend authentication server, and the cost is reduced.

### 5.3. Further Security Analysis

In the following, further security analysis is conducted to show that our scheme can resist common attacks and the correctness is ensured.

#### 5.3.1. Resistance to Impersonation Attack

In the department authentication device setup phase, adversary 𝒜 can impersonate neither *SA*’s authentication device *H_SA_* nor the department authentication device *D_j_*. Why 𝒜 cannot successfully mount an impersonation attack in the department authentication device setup phase is shown as follows. If 𝒜 wants to impersonate *H_SA_* and setup *D_j_*, he/she needs to send *veri*1, *veri*2, and *cparas* to *D_j_*, where *veri*1 = *H*(*R*_1_||*R*_2_||${\mathrm{ID}}_{D_{j}}$||*G_j_*), *veri*2 = *H*(*R*_1_||*R*_2_||${\mathrm{ID}}_{D_{j}}$||*G_j_||S_j_||* (*H*(${\mathrm{ID}}_{A_{1}}{}^{\prime }$), *e*_1_′)||(*H*(${\mathrm{ID}}_{A_{2}}{}^{\prime }$), *e*_2_′)||…||(*H*(${\mathrm{ID}}_{A_{t_{j}}}{}^{\prime }$), $e_{t_{j}}{}^{\prime }$)||(*r_j,_*_1_, *Share_j,_*_1_)||(*r_j,_*_2_, *Share_j,_*_2_)||…||($r_{{j,t}_{j}}$, ${\mathrm{Share}}_{{j,t}_{j}}$)), *cparas* is the ciphertext of (*S_j_*, (*H*(${\mathrm{ID}}_{A_{1}}{}^{\prime }$), *e*_1_′), (*H*(${\mathrm{ID}}_{A_{2}}{}^{\prime }$), *e*_2_′), …, (*H*(${\mathrm{ID}}_{A_{t_{j}}}{}^{\prime }$), $e_{t_{j}}{}^{\prime }$), (*r_j,_*_1_, *Share_j,_*_1_), (*r_j,_*_2_, *Share_j,_*_2_), …, ($r_{{j,t}_{j}}$, ${\mathrm{Share}}_{{j,t}_{j}}$)) encrypted with the encryption key *TK*, and *TK* = *H*(*R*_1_||*R*_2_||*G_j_*||${\mathrm{ID}}_{D_{j}}$). However, it is impossible for 𝒜 to compute correct *veri*1, *veri*2 and *cparas* because the secret *G_j_* is unknown, where *G_j_* = *H*(${\mathrm{ID}}_{D_{j}}$||*Master*_2_). As a result, *D_j_* will detect that the other party is not *H_SA_* when it computes *veri*1′ = *H*(*R*_1_||*R*_2_||${\mathrm{ID}}_{D_{j}}$||*G_j_*) and checks whether *veri*1′ and *veri*1 are equal or not. On the other hand, if 𝒜 wants to impersonate *D_j_* to cheat *H_SA_* and get essential data, he/she will send *R*_2_ to *H_SA_* and get *veri*1, *veri*2, and *cparas*. Unfortunately, 𝒜 does not know *G_j_* such that *TK* cannot be computed. As a result, *cparas* cannot be decrypted to retrieve *S_j_*. Moreover, because of the properties of hash functions, ${\mathrm{ID}}_{D_{j}}$ and other concealed parameters cannot be retrieved from *veri*1 and *veri*2. Consequently, 𝒜 can impersonate neither *H_SA_* nor *D_j_* to threaten the proposed scheme in the department authentication device setup phase.

On the other hand, adversary 𝒜 can impersonate either an executive’s authentication device $H_{A_{i}}$ or *D_j_* in the authentication phase. Why 𝒜 cannot successfully mount an impersonation attack in the authentication phase is shown as follows. If 𝒜 wants to impersonate *D_j_* to cheat $H_{A_{i}}$, 𝒜 needs to send *R*_2_, *PID*, *cshares*, and *check* to $H_{A_{i}}$, where *PID* = *TMS* ⊕ ${\mathrm{ID}}_{D_{j}}$, *check* = *H*(*R*_1_||*R*_2_||(*r_j,_*_1_, *Share_j,_*_1_)||(*r_j,_*_2_, *Share_j,_*_2_)||…||($r_{{j,t}_{j}}$, ${\mathrm{Share}}_{{j,t}_{j}}$)||*MS*||${\mathrm{ID}}_{D_{j}}$||*S_j_*), and *cshares* is the ciphertext of ((*r_j,_*_1_, *Share_j,_*_1_), (*r_j,_*_2_, *Share_j,_*_2_), …, ($r_{{j,t}_{j}}$, ${\mathrm{Share}}_{{j,t}_{j}}$)) with the encryption key *TMS* = *H*(*R*_1_||*R*_2_||*MS*). After receiving *R*_2_, *PID*, *cshares* and *check*, $H_{A_{i}}$ computes *TMS*′ = *H*(*R*_1_||*R*_2_||*MS*), decrypts *cshares* with *TMS*′ to retrieve (*r_j,_*_1_′, *Share_j,_*_1_′), (*r_j,_*_2_′, *Share_j,_*_2_′), …, ($r_{{j,t}_{j}}$′, ${\mathrm{Share}}_{{j,t}_{j}}$′), and uses these shares and (${\mathrm{ID}}_{A_{i}}$, *K_i_*) to obtain *S_j_*′. Then, $H_{A_{i}}$ computes ${\mathrm{ID}}_{D_{j}}$′ = *TMS*′ ⊕ *PID* and *check*′ = *H*(*R*_1_||*R*_2_||(*r_j,_*_1_′, *Share_j,_*_1_′)||(*r_j,_*_2_′, *Share_j,_*_2_′)||…||($r_{{j,t}_{j}}$′, ${\mathrm{Share}}_{{j,t}_{j}}$′)||*MS*||${\mathrm{ID}}_{D_{j}}$′||*S_j_*′) and checks whether *check*′ and *check* are equal or not. Because ${\mathrm{ID}}_{D_{j}}$ is concealed when it is transmitted and only legal *D_j_* knows both ${\mathrm{ID}}_{D_{j}}$ and *S_j_*, only legal *D_j_* can compute *check* to be successfully authenticated by $H_{A_{i}}$ x. That is, 𝒜 cannot impersonate *D_j_* to cheat $H_{A_{i}}$. On the other hand, if 𝒜 wants to impersonate $H_{A_{i}}$ to cheat *D_j_* and obtain the desired rights, 𝒜 needs to send correct *σ*_1_ and *σ*_2_ to *D_j_*, where *σ*_1_* = H*(*R*_1_||*R*_2_||*S_j_*’) ⊕ ${\;\mathrm{ID}}_{A_{i}}$ and *σ*_2_ = ${\mathrm{SK}}_{i}^{{H(\mathrm{ID}}_{A_{i}}{||R}_{1}{||R}_{2}{||S}_{j}{}^{\prime })}$ mod *n*. After receiving *σ*_1_ and *σ*_2_, *D_j_* computes ${\mathrm{ID}}_{A_{i}}$′ = *H*(*R*_1_||*R*_2_||*S_j_*) ⊕ *σ*_1_, uses ${\mathrm{ID}}_{A_{i}}$′ as the index to find the matched (*H*(${\mathrm{ID}}_{A_{i}}{}^{\prime }$), *e_i_*′), and checks whether $g^{{H(\mathrm{ID}}_{A_{i}}{}^{\prime }{||R}_{1}{||R}_{2}{||S}_{j})}$ mod *n* and $\sigma_{2}^{e_{i}{}^{\prime }}$ mod *n* are equal or not. Because ${\mathrm{ID}}_{A_{i}}$ is concealed when it is transmitted and only $H_{A_{i}}$ knows both ${\mathrm{ID}}_{A_{i}}$ and *SK_i_*, only legal $H_{A_{i}}$ can compute correct *σ*_2_ to be successfully authenticated by *D_j_*. Thus, it is impossible for 𝒜 to compute correct *σ*_1_ and *σ*_2_ and cheat *D_j_*. Consequently, 𝒜 can impersonate neither $H_{A_{i}}$ nor *D_j_* to threaten the proposed scheme in the authentication phase.

#### 5.3.2. Resistance to Replay Attack

When adversary 𝒜 eavesdrops and attempts to mount a replay attack and set *D_j_* in the department authentication device setup phase, he/she can send *R*_1_, *veri*1, *veri*2, and *cparas* to *D_j_* of one previous session *D_j_*, where *veri*1 = *H*(*R*_1_||*R*_2_||${\mathrm{ID}}_{D_{j}}$||*G_j_*), *veri*2 = *H*(*R*_1_||*R*_2_||${\mathrm{ID}}_{D_{j}}$||*G_j_||S_j_||* (*H*(${\mathrm{ID}}_{A_{1}}{}^{\prime }$), *e*_1_′)||(*H*(${\mathrm{ID}}_{A_{2}}{}^{\prime }$), *e*_2_′)||…||(*H*(${\mathrm{ID}}_{A_{t_{j}}}{}^{\prime }$), $e_{t_{j}}{}^{\prime }$)||(*r_j,_*_1_, *Share_j,_*_1_)||(*r_j,_*_2_, *Share_j,_*_2_)||…||($r_{{j,t}_{j}}$, ${\mathrm{Share}}_{{j,t}_{j}}$)), *cparas* is the ciphertext of (*S_j_*, (*H*(${\mathrm{ID}}_{A_{1}}{}^{\prime }$), *e*_1_′), (*H*(${\mathrm{ID}}_{A_{2}}{}^{\prime }$), *e*_2_′), …, (*H*(${\mathrm{ID}}_{A_{t_{j}}}{}^{\prime }$), $e_{t_{j}}{}^{\prime }$), (*r_j,_*_1_, *Share_j,_*_1_), (*r_j,_*_2_, *Share_j,_*_2_), …, ($r_{{j,t}_{j}}$, ${\mathrm{Share}}_{{j,t}_{j}}$)) encrypted with the encryption key *TK*, and *TK* = *H*(*R*_1_||*R*_2_||*G_j_*||${\mathrm{ID}}_{D_{j}}$). However, because the random number *R*_2_ is chosen by *D_j_*, *R*_2_ in the present session must differ from that intercepted in the previous session. Thus, the resent *veri*1 must differ from the correct *veri*1′ computed by *D_j_* in the present session, and 𝒜 cannot successfully mount a replay attack in the department authentication device setup phase.

On the other hand, when 𝒜 eavesdrops and attempts to mount a replay attack in the authentication phase, he/she can perform as follows. First, 𝒜 can send *R*_2_, *PID*, *cshares*, and *check* of one previous session to $H_{A_{i}}$, where *PID* = *TMS* ⊕ ${\mathrm{ID}}_{D_{j}}$, *check* = *H*(*R*_1_||*R*_2_||(*r_j,_*_1_, *Share_j,_*_1_)||(*r_j,_*_2_, *Share_j,_*_2_)||…||($r_{{j,t}_{j}}$, ${\mathrm{Share}}_{{j,t}_{j}}$)||*MS*||${\mathrm{ID}}_{D_{j}}$||*S_j_*), and *cshares* is the ciphertext of ((*r_j,_*_1_, *Share_j,_*_1_), (*r_j,_*_2_, *Share_j,_*_2_), …, ($r_{{j,t}_{j}}$, ${\mathrm{Share}}_{{j,t}_{j}}$)) with the encryption key *TMS* = *H*(*R*_1_||*R*_2_||*MS*). However, because the random number *R*_1_ is chosen by $H_{A_{i}}$, *R*_1_ in the present session must differ from that intercepted in the previous session. Thus, *TMS* of the previous session must differ from the correct *TMS*′ computed by $H_{A_{i}}$ in the present session, and $H_{A_{i}}$ cannot retrieve correct ((*r_j,_*_1_, *Share_j,_*_1_), (*r_j,_*_2_, *Share_j,_*_2_), …, ($r_{{j,t}_{j}}$, ${\mathrm{Share}}_{{j,t}_{j}}$)), ${\mathrm{ID}}_{D_{j}}$′ and *S_j_*′. Thereupon, *check*′ computed by $H_{A_{i}}$ in the present session must differ from the resent *check*, and $H_{A_{i}}$ will detect that the other party is not *D_j_*. Second, 𝒜 can send *σ*_1_ and *σ*_2_ of one previous session to *D_j_*, where *σ*_1_* = H*(*R*_1_||*R*_2_||*S_j_*′) ⊕ ${\mathrm{ID}}_{A_{i}}$ and *σ*_2_ = ${\mathrm{SK}}_{i}^{{H(\mathrm{ID}}_{A_{i}}{||R}_{1}{||R}_{2}{||S}_{j}{}^{\prime })}$ mod *n*. Then, *D_j_* uses ${\mathrm{ID}}_{A_{i}}{}^{\prime }$ as the index to find the matched (*H*(${\mathrm{ID}}_{A_{i}}{}^{\prime }$), *e_i_*′) and checks whether $g^{{H(\mathrm{ID}}_{A_{i}}{}^{\prime }{||R}_{1}{||R}_{2}{||S}_{j})}$ mod *n* and $\sigma_{2}^{e_{i}{}^{\prime }}$ mod *n* are equal or not. However, because the random number *R*_2_ is chosen by *D_j_*, *R*_2_ in the present session must differ from that intercepted in the previous session. Thus, ${\mathrm{ID}}_{A_{i}}{}^{\prime }$ computed by *D_j_* in the present session must differ from the correct ${\mathrm{ID}}_{A_{i}}$, such that no matched (*H*(${\mathrm{ID}}_{A_{i}}{}^{\prime }$), *e_i_*′) can be found. From now on, *D_j_* will detect that the other party is not $H_{A_{i}}$. From the above, it is shown that 𝒜 cannot mount a replay attack successfully in the authentication phase, either.

#### 5.3.3. Resistance to Man-in-the-Middle Attack

Man-in-the-middle attack is a type of eavesdropping, where an attacker may intercept, control the exchanged messages, and further capture or manipulate sensitive data without being noticed. When adversary 𝒜 wants to mount a man-in-the-middle attack in our scheme, he/she cannot succeed in either the department authentication device setup phase or authentication phase. How the proposed scheme can defend against a man-in-the-middle attack is shown as follows.

In the department authentication device setup phase, *H_SA_* sends *veri*1, *veri*2, and *cparas* to *D_j_*, where *veri*1 = *H*(*R*_1_||*R*_2_||${\mathrm{ID}}_{D_{j}}$||*G_j_*), *veri*2 = *H*(*R*_1_||*R*_2_||${\mathrm{ID}}_{D_{j}}$||*G_j_*||*S_j_*||(*H*(${\mathrm{ID}}_{A_{1}}{}^{\prime }$), *e*_1_′)||(*H*(${\mathrm{ID}}_{A_{2}}{}^{\prime }$), *e*_2_′)||…||(*H*(${\mathrm{ID}}_{A_{t_{j}}}{}^{\prime }$), $e_{t_{j}}{}^{\prime }$)||(*r_j,_*_1_, *Share_j,_*_1_)||(*r_j,_*_2_, *Share_j,_*_2_)||…||($r_{{j,t}_{j}}$, ${\mathrm{Share}}_{{j,t}_{j}}$)), and *cparas* is the ciphertext of (*S_j_*, (*H*(${\mathrm{ID}}_{A_{1}}{}^{\prime }$), *e*_1_′), (*H*(${\mathrm{ID}}_{A_{2}}{}^{\prime }$), *e*_2_′), …, (*H*(${\mathrm{ID}}_{A_{t_{j}}}{}^{\prime }$), $e_{t_{j}}{}^{\prime }$), (*r_j,_*_1_, *Share_j,_*_1_), (*r_j,_*_2_, *Share_j,_*_2_), …, ($r_{{j,t}_{j}}$, ${\mathrm{Share}}_{{j,t}_{j}}$)). Because of the properties of one-way hash functions, it is hard for 𝒜 to retrieve the unknown parameters, such as ${\mathrm{ID}}_{D_{j}}$, *G_j_* and *S_j_*, from *veri*1 and *veri*2. On the other hand, *cparas* is the ciphertext of (*S_j_*, (*H*(${\mathrm{ID}}_{A_{1}}{}^{\prime }$), *e*_1_′), (*H*(${\mathrm{ID}}_{A_{2}}{}^{\prime }$), *e*_2_′), …, (*H*(${\mathrm{ID}}_{A_{t_{j}}}{}^{\prime }$), $e_{t_{j}}{}^{\prime }$), (*r_j,_*_1_, *Share_j,_*_1_), (*r_j,_*_2_, *Share_j,_*_2_), …, ($r_{{j,t}_{j}}$, ${\mathrm{Share}}_{{j,t}_{j}}$)) with *TK* = *H*(*R*_1_||*R*_2_||*G_j_*||${\mathrm{ID}}_{D_{j}}$). Because *G_j_* and ${\mathrm{ID}}_{D_{j}}$ are unknown, 𝒜 cannot obtain *TK* to decrypt *cparas* to retrieve *S_j_*. Furthermore, 𝒜 cannot control the messages exchanged in the department authentication device setup phase, either.

On the other hand, in the authentication phase, *D_j_* sends *R*_2_, *PID*, *cshares*, and *check* to $H_{A_{i}}$, where *PID* = *TMS* ⊕ ${\mathrm{ID}}_{D_{j}}$, *check* = *H*(*R*_1_||*R*_2_||(*r_j,_*_1_, *Share_j,_*_1_)||(*r_j,_*_2_, *Share_j,_*_2_)||…||($r_{{j,t}_{j}}$, ${\mathrm{Share}}_{{j,t}_{j}}$)||*MS*||${\mathrm{ID}}_{D_{j}}$||*S_j_*), and *cshares* is the ciphertext of ((*r_j,_*_1_, *Share_j,_*_1_), (*r_j,_*_2_, *Share_j,_*_2_), …, ($r_{{j,t}_{j}}$, ${\mathrm{Share}}_{{j,t}_{j}}$)) with *TMS* = *H*(*R*_1_||*R*_2_||*MS*). Then, $H_{A_{i}}$ sends *σ*_1_ and *σ*_2_, where *σ*_1_ = *H*(*R*_1_||*R*_2_||*S_j_*′) ⊕ ${\mathrm{ID}}_{A_{i}}$ and *σ*_2_ = ${\mathrm{SK}}_{i}^{{H(\mathrm{ID}}_{A_{i}}{||R}_{1}{||R}_{2}{||S}_{j}{}^{\prime })}$ mod *n*. Firstly, because ${\mathrm{ID}}_{D_{j}}$ is concealed and not transmitted, 𝒜 cannot retrieve *TMS* from *PID*. Secondly, because of the properties of one-way hash functions, it is hard for 𝒜 to retrieve the unknown parameters, such as *MS*, ${\mathrm{ID}}_{D_{j}}$ and *S_j_*, from *check*. Thirdly, because *MS* is unknown, 𝒜 can neither obtain *TMS* to decrypt *cshares* nor retrieve ${\mathrm{ID}}_{D_{j}}$ from *PID*. Fourthly, because *S_j_* is unknown, it is impossible for 𝒜 to retrieve ${\mathrm{ID}}_{A_{i}}$ from *σ*_1_. Furthermore, 𝒜 cannot control the exchanged messages in the authentication phase, either.

#### 5.3.4. Proof of Correctness

In the initialization phase, the management server *Server* determines *Set*_1_ and *Set*_2_ and initializes a department *C_j_*’s department authentication device *D_j_* for *j* = 1, 2, …, *w*. Then, *Server* confirms the corresponding executives of all departments, computes the authentication data for all executives and departments, stores personal authentication data of the executive *A_i_* in his/her authentication device $H_{A_{i}}$, and stores department authentication data in *SA*’s authentication device *H_SA_*. The fundamental principle of the proposed scheme is only a legal and authorized executive can use his/her authentication device to be successfully authenticated by the corresponding department authentication device while authentication data stored in the executive’s authentication device does not need to be updated even when personnel changes are made. To achieve this goal, *Server* uses (${\mathrm{ID}}_{A_{1}}{}^{\prime }$, *K*_1_′), (${\mathrm{ID}}_{A_{2}}{}^{\prime }$, *K*_2_′), …, (${\mathrm{ID}}_{A_{t_{j}}}{}^{\prime }$, $K_{t_{j}}{}^{\prime }$) of executives *A*_1_′, *A*_2_′, …, $A_{t_{j}}{}^{\prime }$ who are authorized to manage or inspect *C_j_*, and (0, *S_j_*) to obtain the polynomial *P_j_*(*x*) = $a_{t_{j}}x^{t_{j}}$ + $a_{t_{j}-1}x^{t_{j}-1}$+ … + *a*_1_*x* + *S_j_* mod *n*, where *t_j_* is the number of authorized executives who can manage or inspect *C_j_*, {*A*_1_′, *A*_2_′, …, $A_{t_{j}}{}^{\prime }$} ⊆ {*A_i_*|*i* = 1, 2, …, *m*}, *P_j_*(0) = *S_j_*, *P_j_*(${\mathrm{ID}}_{A_{1}}{}^{\prime }$) = *K*_1_′, *P_j_*(${\mathrm{ID}}_{A_{2}}{}^{\prime }$) = *K*_2_′, …, and *P_j_*(${\mathrm{ID}}_{A_{t_{j}}}{}^{\prime }$) = $\;K_{t_{j}}{}^{\prime }$. After obtaining the polynomial *P_j_*(*x*), *Server* randomly generates *r_j,_*_1_, *r_j,_*_2_, …, $r_{{j,t}_{j}}$ and computes *Share_j,_*_1_ = *P_j_*(*r_j,_*_1_), *Share_j,_*_2_ = *P_j_*(*r_j,_*_2_), …, ${\mathrm{Share}}_{{j,t}_{j}}$ = *P_j_*($r_{{j,t}_{j}}$), where *r_j,_*_1_, *r_j,_*_2_, …, $r_{{j,t}_{j}}$ are less than min(*p*, *q*), *r_j,_*_1_, *r_j,_*_2_, …, $r_{{j,t}_{j}}$ are different from each other and *r_j,_*_1_, *r_j,_*_2_, …, $r_{{j,t}_{j}}$ are different from ${\mathrm{ID}}_{A_{1}}{}^{\prime }$, ${\mathrm{ID}}_{A_{2}}{}^{\prime }$, …, ${\mathrm{ID}}_{A_{t_{j}}}{}^{\prime }$. *Server* stores *H*(.), *n*, *g*, *MS*, ${\mathrm{ID}}_{D_{j}}$, *G_j_*, (*H*(${\mathrm{ID}}_{A_{1}}{}^{\prime }$), *e*_1_′), (*H*(${\mathrm{ID}}_{A_{2}}{}^{\prime }$), *e*_2_′), …, (*H*(${\mathrm{ID}}_{A_{t_{j}}}{}^{\prime }$), $e_{t_{j}}{}^{\prime }$), (*r_j,_*_1_, *Share_j,_*_1_), (*r_j,_*_2_, *Share_j,_*_2_), …, ($r_{{j,t}_{j}}$, ${\mathrm{Share}}_{{j,t}_{j}}$) and *S_j_* in the system administrator *SA*’s authentication device *H_SA_*, where *j* = 1, 2, …, *w*.

Later, *SA* can initialize the authentication data in *C_j_*’s department authentication device *D_j_* in the department authentication device setup phase such that *S_j_*, (*H*(${\mathrm{ID}}_{A_{1}}{}^{\prime }$), *e*_1_′), (*H*(${\mathrm{ID}}_{A_{2}}{}^{\prime }$), *e*_2_′), …, (*H*(${\mathrm{ID}}_{A_{t_{j}}}{}^{\prime }$), $e_{t_{j}}{}^{\prime }$), (*r_j,_*_1_, *Share_j,_*_1_), (*r_j,_*_2_, *Share_j,_*_2_), …, ($r_{{j,t}_{j}}$, ${\mathrm{Share}}_{{j,t}_{j}}$) are stored in *D_j_*. Thereupon, in the authentication phase, $H_{A_{i}}$ uses (*r_j,_*_1_′, *Share_j,_*_1_′), (*r_j,_*_2_′, *Share_j,_*_2_′), …, ($r_{{j,t}_{j}}$′, ${\mathrm{Share}}_{{j,t}_{j}}$′) and (${\mathrm{ID}}_{A_{i}}$, *K_i_*) to set the parameters *q*_0_ = ${\mathrm{ID}}_{A_{i}}$, *Q*_0_ = *K_i_*, *q*_1_ = *r_j,_*_1_′, *Q*_1_ = *Share_j,_*_1_′, *q*_2_ = *r_j,_*_2_′, *Q*_2_ = *Share_j,_*_2_′, …, $q_{t_{j}}$ = $r_{{j,t}_{j}}$′, $Q_{t_{j}}$ = ${\mathrm{Share}}_{{j,t}_{j}}$′. Then, $H_{A_{i}}$ computes *S_j_*′ = $\sum_{u=0}^{t_{j}}J_{u}{\times Q}_{u}$ mod *n*, where $J_{u}=\prod_{q_{b}\neq q_{u}}\frac{{-q}_{b}}{q_{u}{-q}_{b}}$.

Because *t_j_* is the number of authorized executives who can manage or inspect *C_j_* and the polynomial *P_j_*(*x*) of degree *t_j_*, *Server* obtains *P_j_*(*x*) with (${\mathrm{ID}}_{A_{1}}{}^{\prime }$, *K*_1_′), (${\mathrm{ID}}_{A_{2}}{}^{\prime }$, *K*_2_′), …, (${\mathrm{ID}}_{A_{t_{j}}}{}^{\prime }$, $K_{t_{j}}{}^{\prime }$) and (0, *S_j_*), where *P_j_*(0) = *S_j_*, *P_j_*(${\mathrm{ID}}_{A_{1}}{}^{\prime }$) = *K*_1_′, *P_j_*(${\mathrm{ID}}_{A_{2}}{}^{\prime }$) = *K*_2_′, …, and *P_j_*(${\mathrm{ID}}_{A_{t_{j}}}{}^{\prime }$) = $\;K_{t_{j}}{}^{\prime }$. After *P_j_*(*x*) is obtained, *Share_j,_*_1_, *Share_j,_*_2_, …, ${\mathrm{Share}}_{{j,t}_{j}}$ can be easily computed, where *Share_j,_*_1_ = *P_j_*(*r_j,_*_1_), *Share_j,_*_2_ = *P_j_*(*r_j,_*_2_), …, ${\mathrm{Share}}_{{j,t}_{j}}$ = *P_j_*($r_{{j,t}_{j}}$). An authorized executive *A_i_*’s $H_{A_{i}}$ with (${\mathrm{ID}}_{A_{i}}$, *K_i_*) ∈ {(${\mathrm{ID}}_{A_{1}}{}^{\prime }$, *K*_1_′), (${\mathrm{ID}}_{A_{2}}{}^{\prime }$, *K*_2_′), …, (${\mathrm{ID}}_{A_{t_{j}}}{}^{\prime }$, $K_{t_{j}}{}^{\prime }$)} can retrieve *P_j_*(*x*) of degree *t_j_*, when (*r_j,_*_1_, *Share_j,_*_1_), (*r_j,_*_2_, *Share_j,_*_2_), …, ($r_{{j,t}_{j}}$, ${\mathrm{Share}}_{{j,t}_{j}}$) are obtained. On the other hand, because the constant term *S_j_* of *P_j_*(*x*) is the dedicated authentication code for *D_j_*, $H_{A_{i}}$ utilizes the Lagrange interpolation formula to compute *S_j_*′ = $\sum_{u=0}^{t_{j}}J_{u}{\times Q}_{u}$ mod *n* to retrieve *S_j_* only, instead of the polynomial *P_j_*(*x*), where $J_{u}=\prod_{q_{b}\neq q_{u}}\frac{{-q}_{b}}{q_{u}{-q}_{b}}$. From the above, the correctness can be ensured.

## 6. Performance Evaluation and Further Discussion

This section evaluates the performance of the proposed scheme. The test was implemented in Python 3 on a personal computer with Intel (R) Core (TM) i7-9750H 2.60 GHz CPU, 16.0 GB RAM, and a 64-bits Windows 10 operating system. The analysis was divided into two categories: (1) communication cost and (2) computational cost. To ensure security, in the evaluation, SHA-256 and AES with a block size of 128 bits and a key length of 256 bits are adopted, and the lengths of *p*, *q*, and *n* are 1024-bit, 1024-bit, and 2048-bit, respectively. In Section 6.1, we evaluate the communication costs of the department authentication device setup and authentication phases in which messages are transmitted. Section 6.2 analyzes the computational costs for the initialization, department authentication device setup and authentication phases. In Section 6.3, further discussion is presented.

### 6.1. Analysis of Communication Cost

The proposed user authentication scheme can be regarded as an application layer protocol. How to transmit data between devices and fix bit errors are defined by transmission standards such as Bluetooth. To analyze the communication cost of the proposed scheme, the extra data transmission resulting from transmission standards is not taken into consideration. The communication cost for one phase is the number of bits of messages exchanged in this phase. In the proposed scheme, messages are exchanged in only the department authentication device setup phase and authentication phase, so communication costs for these two phases are evaluated. The communication cost for the proposed scheme is shown in Table 2. For generality, let *t_j_* represent the total number of executives who can manage and inspect *C_j_*. In the department authentication device setup phase, three messages are exchanged. The first message contains a 2048-bit random number *R*_1_. Then, the second message contains another 2048-bit random number *R*_2_. The third message contains *cparas* and two hash values, *veri*1 and *veri*2. Because *cparas* is the ciphertext of {*S_j_*, (*H*(${\mathrm{ID}}_{A_{1}}{}^{\prime }$), *e*_1_′), (*H*(${\mathrm{ID}}_{A_{2}}{}^{\prime }$), *e*_2_′), …, (*H*(${\mathrm{ID}}_{A_{t_{j}}}{}^{\prime }$), $e_{t_{j}}{}^{\prime }$), (*r_j,_*_1_, *Share_j,_*_1_), (*r_j,_*_2_, *Share_j,_*_2_), …, ($r_{{j,t}_{j}}$, ${\mathrm{Share}}_{{j,t}_{j}}$)}, its size is 2048 + *t_j_* × (256 + 2048) + *t_j_* × (2048 + 2048) = (2048 + 6400*t_j_*) bits. Therefore, the size of the messages transmitted in the department authentication device setup phase is (6656 + 6400*t_j_*) bits.

In the authentication phase, three messages are exchanged. The first message contains one 2048-bit random number *R*_1_. The second message contains one 2048-bit random number *R*_2_, *PID*, *cshares*, and one hash value *check*. Because *cshares* is the ciphertext of ((*r_j,_*_1_, *Share_j,_*_1_), (*r_j,_*_2_, *Share_j,_*_2_), …, ($r_{{j,t}_{j}}$, ${\mathrm{Share}}_{{j,t}_{j}}$)), its size is (2048 + 2048) × *t_j_* = 4096*t_j_* bits. The size of *PID* is 256 bits because *PID* = *TMS* ⊕ ${\mathrm{ID}}_{D_{j}}$. The size of *check* is 256 bits. So, the size of the second message is 2048 + 256 + *t_j_* × (2048 + 2048) + 256 = (2560 + 4096*t_j_*) bits. The third message contains *σ*_1_ and *σ*_2_. The size of *σ*_1_ is 256 bits because *σ*_1_ = *H*(*R*_1_||*R*_2_||*S_j_*’) ⊕ ${\mathrm{ID}}_{A_{i}}$. Because the length of *n* is 2048 bits, the size of *σ*_2_ = ${\mathrm{SK}}_{i}^{{H(\mathrm{ID}}_{A_{i}}{||R}_{1}{||R}_{2}{||S}_{j}{}^{\prime })}$ mod *n* is also 2048 bits. Thus, the size of the third message is 256+2048 = 2304 bits. The size of the messages transmitted in the authentication phase is 2048 + 2560 + 4096*t_j_* + 2304 = (6912 + 4096*t_j_*) bits. The total communication cost of the proposed scheme is (13,568 + 10,496*t_j_*) bits.

### 6.2. Analysis of Computational Cost

**Figure 6:**
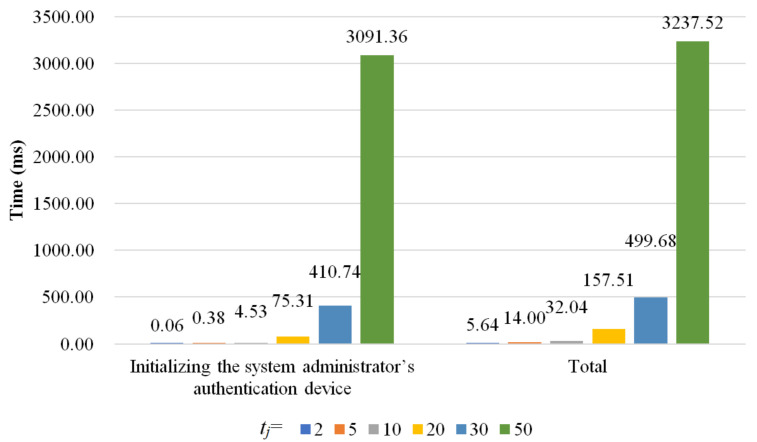
The computational costs for the initialization phase.

**Figure 7:**
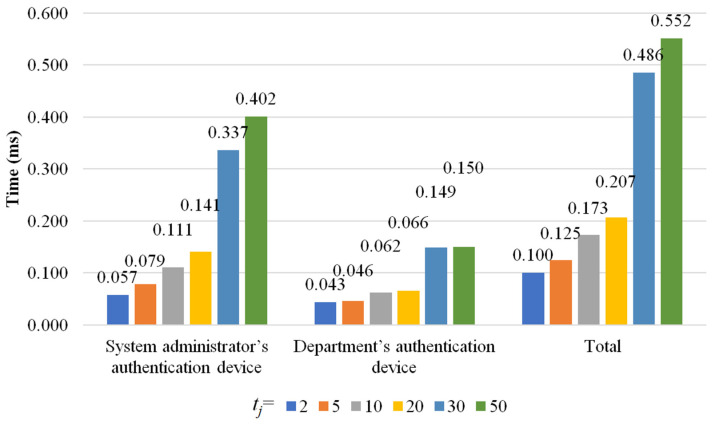
The computational costs for the department authentication device setup phase.

**Figure 8:**
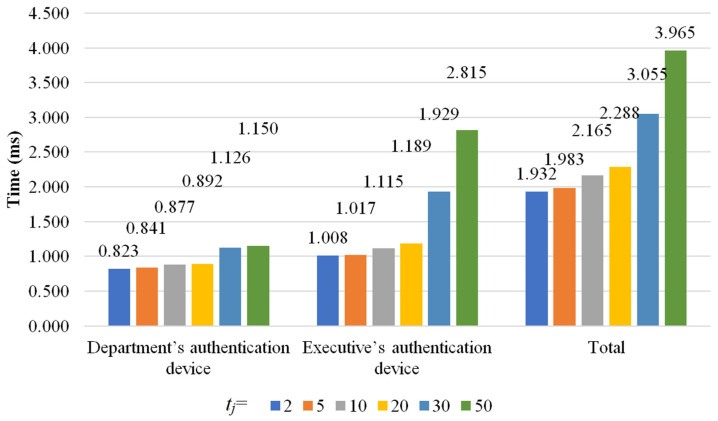
The computational costs for the authentication phase.

There are four phases in the proposed scheme: initialization phase, department authentication device setup phase, authentication phase, and authentication data update phase. Because the authentication data update phase is similar to the initialization phase, we simulate the initialization phase, department authentication device setup phase, and authentication phase to evaluate the computational cost of the proposed scheme. In the simulation, *t_j_* denotes the total number of executives who can manage and inspect *C_j_*, and *t_j_* ∈ {2, 5, 10, 20, 30, 50}. To eliminate the influence of unpredictable factors and make the evaluation essential, we run the simulation 1000 times and compute the average computational costs. The computational costs for the initialization phase, department authentication device setup phase, and authentication phase are shown in Figure 6, Figure 7 and Figure 8, respectively.

In the initialization phase, *Server* performs three tasks: (1) initializing the department’s authentication device *D_j_*, (2) initializing the executive’s authentication device $H_{A_{i}}$, and (3) initializing the system administrator’s authentication device *H_SA_*. In the first task, *Server* computes *G_j_* = *H*(${\mathrm{ID}}_{D_{j}}$||*Master*_2_). The computational cost of this task is independent of *t_j_*, and it takes 0.00720 milliseconds. In the second task, the server computes *K_i_*, *d_i_*, and *SK_i_* for the executive’s authentication device $H_{A_{i}}$. Similar to the first task, the computational cost of the second task is independent of *t_j_*, and initializing $H_{A_{i}}$ takes 3.09 milliseconds. *Server* computes ${\mathrm{Share}}_{{j,t}_{j}}$ for the system administrator’s authentication device *H_SA_* with the polynomial *P_j_*(*x*) in the third task. There exists a positive correlation between the degree of the polynomial *P_j_*(*x*) and *t_j_*. When *t_j_* increases, both the degree of the polynomial *P_j_*(*x*) and time required to obtain the polynomial increase. Because *t_j_* varies and *t_j_* ∈{2, 5, 10, 20, 30, 50}, the computational costs for the third task are 0.0600 milliseconds, 0.384 milliseconds, 4.53 milliseconds, 75.31 milliseconds, 411 milliseconds, and 3090 milliseconds, respectively. To summarize, the total computational costs for the initialization phase with *t_j_* ∈{2, 5, 10, 20, 30, 50} are 5.64 milliseconds, 14.0 milliseconds, 32.0 milliseconds, 156 milliseconds, 500 milliseconds, and 3240 milliseconds, respectively.

In the department authentication device setup phase, both the system administrator’s authentication device *H_SA_* and the department’s authentication device *D_j_* are involved. *H_SA_* computes *veri*1 and *veri*2 and encrypts {*S_j_*, (*H*(${\mathrm{ID}}_{A_{1}}{}^{\prime }$), *e*_1_′), (*H*(${\mathrm{ID}}_{A_{2}}{}^{\prime }$), *e*_2_′), …, (*H*(${\mathrm{ID}}_{A_{t_{j}}}{}^{\prime }$), $e_{t_{j}}{}^{\prime }$), (*r_j,_*_1_, *Share_j,_*_1_), (*r_j,_*_2_, *Share_j,_*_2_), …, ($r_{{j,t}_{j}}$, ${\mathrm{Share}}_{{j,t}_{j}}$)} to get *cparas* with AES, where *veri*2 is the hash value of (*R*_1_||*R*_2_||${\mathrm{ID}}_{D_{j}}$||*G_j_*||*S_j_*||(*H*(${\mathrm{ID}}_{A_{1}}{}^{\prime }$), *e*_1_′)||(*H*(${\mathrm{ID}}_{A_{2}}{}^{\prime }$), *e*_2_′)||…||(*H*(${\mathrm{ID}}_{A_{t_{j}}}{}^{\prime }$), $e_{t_{j}}{}^{\prime }$)||(*r_j,_*_1_, *Share_j,_*_1_)||(*r_j,_*_2_, *Share_j,_*_2_)||…||($r_{{j,t}_{j}}$, ${\mathrm{Share}}_{{j,t}_{j}}$)). Thus, when *t_j_* increases, the time needed for computing *veri*2 and obtaining *cparas* will increase. For *t_j_* ∈ {2, 5, 10, 20, 30, 50}, *H_SA_* spends 0.0573 milliseconds, 0.0787 milliseconds, 0.111 milliseconds, 0.141 milliseconds, 0.337 milliseconds, and 0.402 milliseconds on computations, respectively.

On the other hand, *veri*1′, *TK*’, and *veri*2′ are computed by *D_j_* in the department authentication device setup phase. *D_j_* decrypts *cparas* to obtain {*S_j_*, (*H*(${\mathrm{ID}}_{A_{1}}{}^{\prime }$), *e*_1_′), (*H*(${\mathrm{ID}}_{A_{2}}{}^{\prime }$), *e*_2_′), …, (*H*(${\mathrm{ID}}_{A_{t_{j}}}{}^{\prime }$), $e_{t_{j}}{}^{\prime }$), (*r_j,_*_1_, *Share_j,_*_1_), (*r_j,_*_2_, *Share_j,_*_2_), …, ($r_{{j,t}_{j}}$, ${\mathrm{Share}}_{{j,t}_{j}}$)}. When *t_j_* increases, the time needed for computing *veri*2′ and decrypting *cparas* will increase. For *t_j_* ∈ {2, 5, 10, 20, 30, 50}, *D_j_* spends 0.0430 milliseconds, 0.0460 milliseconds, 0.0623 milliseconds, 0.0655 milliseconds, 0.149 milliseconds, and 0.150 milliseconds, respectively.

Thus, for *t_j_* ∈ {2, 5, 10, 20, 30, 50}, the total computational costs for the department authentication device setup phase are 0.100 milliseconds, 0.125 milliseconds, 0.173 milliseconds, 0.207 milliseconds, 0.486 milliseconds, and 0.552 milliseconds, respectively.

In the authentication phase, *D_j_* and $H_{A_{i}}$ authenticate each other. *D_j_* computes *TMS*, *PID* and *check* and encrypt ((*r_j,_*_1_, *Share_j,_*_1_), (*r_j,_*_2_, *Share_j,_*_2_), …, ($r_{{j,t}_{j}}$, ${\mathrm{Share}}_{{j,t}_{j}}$)) to get *cshares* with AES. *D_j_* has to retrieve ${\mathrm{ID}}_{A_{i}}$′ and compute $\sigma_{2}^{e_{i}{}^{\prime }}$ mod *n* with the matched *e*_i_′ after receiving the message sent from $H_{A_{i}}$. *check* is the hash value of ((*R*_1_||*R*_2_||(*r_j,_*_1_, *Share_j,_*_1_)||(*r_j,_*_2_, *Share_j,_*_2_)||…||($r_{{j,t}_{j}}$, ${\mathrm{Share}}_{{j,t}_{j}}$)||*MS*||${\mathrm{ID}}_{D_{j}}$||*S_j_*)), so the time needed to compute *check* is positively correlated to *t_j_*. Similarly, when *t_j_* increases, the time needed for encryption will also increase. Thus, for *t_j_* ∈ {2, 5, 10, 20, 30, 50}, *D_j_* spends 0.823 milliseconds, 0.841 milliseconds, 0.877 milliseconds, 0.893 milliseconds, 1.13 milliseconds, and 1.15 milliseconds in the authentication phase, respectively.

On the other hand, $H_{A_{i}}$ uses AES to decrypt *cshares* after computing *TMS*’, *S_j_*’, ${\mathrm{ID}}_{D_{j}}$′, *check*′, *σ*_1_ and *σ*_2_ in the authentication phase. *check*′ is the hash value of ((*R*_1_||*R*_2_||(*r_j,_*_1_′, *Share_j,_*_1_′)||(*r_j,_*_2_′, *Share_j,_*_2_′)||…||($r_{{j,t}_{j}}$′, ${\mathrm{Share}}_{{j,t}_{j}}$′)||*MS*||${\mathrm{ID}}_{D_{j}}$′||*S_j_*′)), so the time needed to compute *check* is positively correlated to *t_j_*. Similarly, when *t_j_* increases, the time needed for decryption will also increase. In the proposed scheme, Lagrange interpolation is utilized to compute *S_j_*′, so *t_j_* is also positively correlated to the time needed to compute *S_j_*′. That is, the larger *t_j_* is, the longer it takes to compute *S_j_*′. Then, for *t_j_* ∈{2, 5, 10, 20, 30, 50}, $H_{A_{i}}$ spends 1.01 milliseconds, 1.02 milliseconds, 1.12 milliseconds, 1.19 milliseconds, 1.93 milliseconds, and 2.82 milliseconds in the authentication phase, respectively.

Thus, for *t_j_* ∈ {2, 5, 10, 20, 30, 50}, the total computational costs for the authentication phase are 1.93 milliseconds, 1.98 milliseconds, 2.17 milliseconds, 2.29 milliseconds, 3.05 milliseconds, and 3.96 milliseconds, respectively.

By the above analysis, it is ensured that the proposed scheme can ensure efficiency and be applied in real-time applications because the time for authentication is far less than one second. On the other hand, although the proposed scheme is designed to help an executive to be authenticated by the department authentication device, it can also be utilized for access control of small-sized enterprises/facilities/apartment complexes while workers/members/residents instead of executives are authenticated.

### 6.3. Further Discussion

In this subsection, we demonstrate the unpredictable factors encountered when we run the simulation. As shown in the previous analysis, many parameters are positively correlated to *t_j_*. In ideal circumstances, the computational costs of computing these parameters should be proportional to *t_j_*. However, after the simulation is run, the outcome is different from that expected. With further analysis, three unpredictable factors that may affect the simulation are found. The details are as follows.

#### 6.3.1. Data Type Conversion

In our proposed system, over 100 parameters are used to compute variables. And data type conversion of these parameters and variables may impact the computational cost. In the authentication phase, for instance, *D_j_* has to compute *check* = *H*(*R*_1_||*R*_2_||(*r_j,_*_1_, *Share_j,_*_1_)||(*r_j,_*_2_, *Share_j,_*_2_)||…||($r_{{j,t}_{j}}$, ${\mathrm{Share}}_{{j,t}_{j}}$)||*MS*||${\mathrm{ID}}_{D_{j}}$||*S_j_*), where all input parameters and variables of the one-way hash function are integers. Integers cannot be concatenated directly, so data type conversion is needed to convert the integer to a string. Moreover, the time for data type conversion is neither constant nor linear. Consequently, data type conversion is an unpredictable factor that may influence the computational cost.

#### 6.3.2. Insufficient Memory

In the initialization phase, the management server *Server* obtains the polynomial *P_j_*(*x*) = $a_{t_{j}}x^{t_{j}}$ + $a_{t_{j}-1}x^{t_{j}-1}$ + … + *a*_1_*x* + *S_j_* mod *n* and computes *Share_j,_*_1_ = *P_j_*(*r_j,_*_1_), *Share_j,_*_2_ = *P_j_*(*r_j,_*_2_), …, ${\mathrm{Share}}_{{j,t}_{j}}$ = *P_j_*($r_{{j,t}_{j}}$). All *Share*’s computed by the polynomial are integers, and they represent the sum of parameters and variables. At first, Python allocates a small amount of memory to store the variable *Share*. However, when the size of *Share* increases, the allocated memory is insufficient. This results in Python having to allocate more memory to store *Share*. This approach increases the computational cost in the simulation. Consequently, insufficient memory is another unpredictable factor that may influence the computational cost.

#### 6.3.3. Number System Conversion

When one operation manipulates two or more numbers of different or undesired bases, number system conversion is needed. In the authentication phase, for instance, the executive’s authentication device $H_{A_{i}}$ computes *σ*_1_ = *H*(*R*_1_||*R*_2_||*S_j_*’) ⊕ ${\mathrm{ID}}_{A_{i}}$, where *σ*_1_ is computed with a hash value and an identity. Hash values are hexadecimal numbers, and all identities are decimal numbers after the data type conversion. That is, before *σ*_1_ is computed, both *H*(*R*_1_||*R*_2_||*S_j_*’) and ${\mathrm{ID}}_{A_{i}}$ need to be converted to binary numbers. However, number system conversion may increase the computational cost. Moreover, the time for number system conversion is neither constant nor linear. Thus, number system conversion is also an unpredictable factor that may influence the computational cost.

## 7. Conclusions

This paper proposes an offline user authentication system that ensures non-repudiation and anonymity. With the proposed scheme, management can be easily conducted even when personnel changes are made. We show that the proposed scheme satisfies the desired requirements and can resist common attacks. Additionally, we evaluate its performance by analyzing communication cost and computational cost, and further discussion shows three unpredictable factors that may affect the computational cost in the simulation. By the analysis and evaluation mentioned above, it is ensured that the proposed offline user authentication system can be applied to real-time applications that possess the same requirements in the real world.

## Figures and Tables

**Table 1:** Notations used in the proposed user authentication scheme.

Notation	Definitions
*Server*	Management server
*Master*_1_ *, Master*_2_	*Server*’s secret keys for generating essential parameters for authentication devices
*MS*	System secret key for checking the integrity of the transmitted data and generating session keys to protect the transmitted data
*SA*	System administrator
*H_SA_*	*SA*’s authentication device
*m*	The total amount of executives of management personnel in the system
*A_i_*	The *i*th executive
*Set*_1_	The set of executives in the system, where *Set*_1_ = {*A_i_*|*i* is in [1, *m*]} and|*Set*_1_| = *m*
${\mathrm{ID}}_{A_{i}}$	*A_i_*’s unique identity
$H_{A_{i}}$	*A_i_*’s authentication device
*w*	The total amount of departments in the system
*C_j_*	The *j*th department
*Set*_2_	The set of departments in the system, where *Set*_2_ = {*C_j_*|*j* is in [1, *w*]} and|*Set*_2_| = *w*
*t_j_*	The number of executives *C_j_*, where *t_j_* >= 1
*D_j_*	*C_j_*’s department authentication device
${\mathrm{ID}}_{D_{j}}$	*D_j_*’s unique identity
*H*(.)	One-way hash function, where *H*: {0, 1}* → {0, 1}*^l^* and *l* is the length of its output
*p, q*	Two large prime integers chosen by *Server* and secretly kept by *Server*, where *p* > 1*^l^*
*n*	System public parameter, where *n* = *p* × *q*
*g*	The primitive root modulo *n*
||	Concatenation operator
⊕	XOR operator

**Table 2:** The communication cost for the proposed scheme.

Phase	Messages	Communication Cost (Bits)
Department authentication device setup phase	3	6656 + 6400*t_j_*
Authentication phase	3	6912+ 4096*t_j_*
Total	6	13,568 + 10,496*t_j_*

## Data Availability

Not applicable.
